# Structural and biomedical investigations of novel ruthenium schiff base complexes

**DOI:** 10.1038/s41598-025-03147-9

**Published:** 2025-05-27

**Authors:** Ramadan M. Ramadan, Hadeel H. El-Shalakany, Mostafa A. Sayed

**Affiliations:** Chemistry Department, Faculty of Science, Ain Shams University, Cairo, 11566 Egypt

**Keywords:** Ru(III) complexes, Schiff base ligands, Molecular docking, DFT analysis, Biological activity, Chemistry, Coordination chemistry

## Abstract

Ruthenium(III) complexes with Schiff base ligands bearing diverse functional groups remain extensively underexplored, despite their promising potential in therapeutic applications. To address this gap, we designed and synthesized a new series of mononuclear octahedral Ru(III) complexes with the general formula [**RuL**^**1–3**^], where **L**^**1**^, **L**^**2**^, and **L**^**3**^ are deprotonated Schiff bases derived from functionalized aromatic precursors. These complexes were characterized through a suite of physicochemical and spectroscopic techniques, including FT-IR, ^1^H-NMR, UV-Vis spectroscopy, mass spectrometry, TGA, and elemental analysis, to confirm their structural features and coordination environment. To complement experimental findings, density functional theory (DFT/B3LYP) calculations were conducted, revealing stable, distorted octahedral geometries and supporting the proposed molecular configurations. Building upon the structural insights, we evaluated the biological activity of the complexes through in vitro cytotoxicity assays against HCT-116 (colorectal), MCF-7 (breast), and HepG2 (liver) cancer cell lines. Among them, **RuL**^**2**^ exhibited the most potent activity against HCT-116 (IC₅₀ = 4.97 µg/mL), comparable to the standard drug Vinblastine. Finally, molecular docking simulations were employed to investigate the interaction of these complexes with key biological targets from *Escherichia coli* (PDB IDs: 4BJP and 1BNA), offering further insights into their potential modes of action. Together, these results demonstrate the importance of ligand design in tuning the coordination behavior and bioactivity of ruthenium complexes, highlighting their promise in anticancer and antimicrobial drug development.

**Supplementary Information:**

The online version contains supplementary material available at 10.1038/s41598-025-03147-9.

## Introduction

Schiff bases, characterized by the imine (‒C = N‒) functional group, are widely utilized in coordination chemistry due to their ability to form stable and structurally diverse metal complexes^1–3^. Typically synthesized through the condensation of primary amines with carbonyl compounds, Schiff bases offer versatile donor atoms—commonly nitrogen (N), oxygen (O), and sulfur (S)—that promote strong chelation with metal centers^4–7^. The nature and arrangement of these donor atoms significantly influence the geometry, stability, and reactivity of the resulting complexes, as well as their interaction with biological targets such as DNA and proteins^8–12^. Incorporation of electron-donating (e.g., hydroxyl, methoxy) or electron-withdrawing (e.g., nitro, chloro) substituents into the Schiff base framework further tunes the electronic properties of metal complexes, impacting their redox behavior, lipophilicity, and biological activity^13–15^. Owing to these tunable features, Schiff base complexes have demonstrated a wide spectrum of pharmacological properties, including antibacterial, antifungal, antioxidant, antidiabetic, and anticancer effects^16–18^. Furthermore, the biological function of Schiff base metal complexes is strongly influenced by the specific donor atoms present and their three-dimensional configuration^19,20^.

Cancer remains a leading cause of mortality worldwide, and current chemotherapeutic agents, especially platinum-based drugs such as cisplatin, are associated with severe side effects and the emergence of drug resistance^21^. These limitations necessitate the search for new metal-based therapeutics with enhanced selectivity and lower toxicity. Ruthenium complexes have emerged as promising candidates due to their favorable pharmacokinetics, redox versatility, and ability to engage in multiple modes of action, including DNA binding and protein inhibition^22–25^. Compared to platinum-based medications, ruthenium complexes have demonstrated remarkable antitumor activity, along with improved efficacy, reduced drug resistance, and less toxicity^26^. Ruthenium complexes incorporating Schiff base ligands have shown remarkable potential in biological applications, notably in antioxidant, antimicrobial, and anticancer activities^27,28^. The octahedral coordination geometry favored by both Ru(II)/Ru(III) is a key factor in the effectiveness of these ruthenium complexes^29^. Particularly, ruthenium(III) complexes with Schiff base ligands have shown potent cytotoxicity against various cancer cell lines and notable antimicrobial activity^30,31^.

The biological performance of these complexes is highly dependent on the structural and electronic characteristics of the ligand. Functional groups such as hydroxyl, methoxy, and nitro have been reported to enhance DNA-binding affinity, cellular uptake, and selective toxicity toward cancer cells^32^. Moreover, Schiff base ligands significantly influence the spatial configuration and redox behavior of ruthenium centers, thus affecting their biological mechanisms of action^33,34^. Previous studies have shown that Schiff base Ru(III) complexes exhibit notable cytotoxicity against various cancer cell lines, with IC₅₀ values often comparable to or better than standard drugs^24,35–38^. Despite the therapeutic potential of ruthenium(III) complexes, their coordination chemistry and biomedical applications remain underexplored, particularly with Schiff base ligands bearing diverse functional groups.

In this study, we report on the synthesis and comprehensive investigation of three novel mononuclear ruthenium(III) complexes bearing Schiff base ligands HL^1^, HL^2^, and H_2_L^3^. These ligands were strategically selected for their distinct functional groups—nitro, chloro, methoxy, and hydroxyl—which are known to influence both coordination behavior and bioactivity. The study encompasses physicochemical characterization, DFT-based structural modeling, and evaluation of anticancer and antimicrobial properties. Cytotoxic effects were assessed against HepG2 (liver), MCF-7 (breast), and HCT-116 (colon) cancer cell lines, while molecular docking studies provided further insight into the complexes’ interactions with biomolecular targets. This work aims to elucidate the structure–activity relationships of ruthenium Schiff base complexes and contribute to the development of new metal-based agents in medicinal inorganic chemistry.

## Experimental

### Materials

RuCl_3_, 2-amino-3-hydroxypyridine, 2-chloro-5-nitrobenzaldehyde, 2-hydroxy-3-methoxybenzaldehyde and 2-amino-4,6-dimethylpyrimidine were obtained from Sigma-Aldrich.

### The physicochemical measurements

Fourier transform infrared (FT-IR) spectra were collected using a Shimadzu FT-IR model 8101 spectrophotometer, with KBr pellets, over the wavelength range of 4000 to 400 cm⁻¹. The conductivity of the ruthenium complexes was measured in 1 × 10⁻³ M solutions of DMF at room temperature using a JENWAY 4320 conductivity meter. The UV-vis spectra of the ligands and their complexes (1 × 10^− 5^ M) were performed by using a Shimadzu 1800 UV-Vis spectrophotometer. All synthesized compounds underwent elemental analysis (C, H, N) using a PerkinElmer-2400 CHN analyzer to determine their elemental composition. This analysis served as a crucial step in validating the accuracy of the proposed molecular formula for each synthesized compound. A Bruker apparatus (Munich, Germany) was used to carry out the proton nuclear magnetic resonance (^1^H-NMR) measurements using DMSO-*d6*. Mass spectra were executed using the EI technique at 70 eV via MS-5988 GC-MS Hewlett-Packard equipment. A Shimadzu Corporation 60 H analyzer was used for the thermogravimetric (TG) measurements. The samples were heated in air at a constant heating rate of 10 °C per minute until a maximum temperature of 1000 °C was achieved.

### Syntheses

#### Synthesis of Imine schiff base ligands

**Fig. 1:**
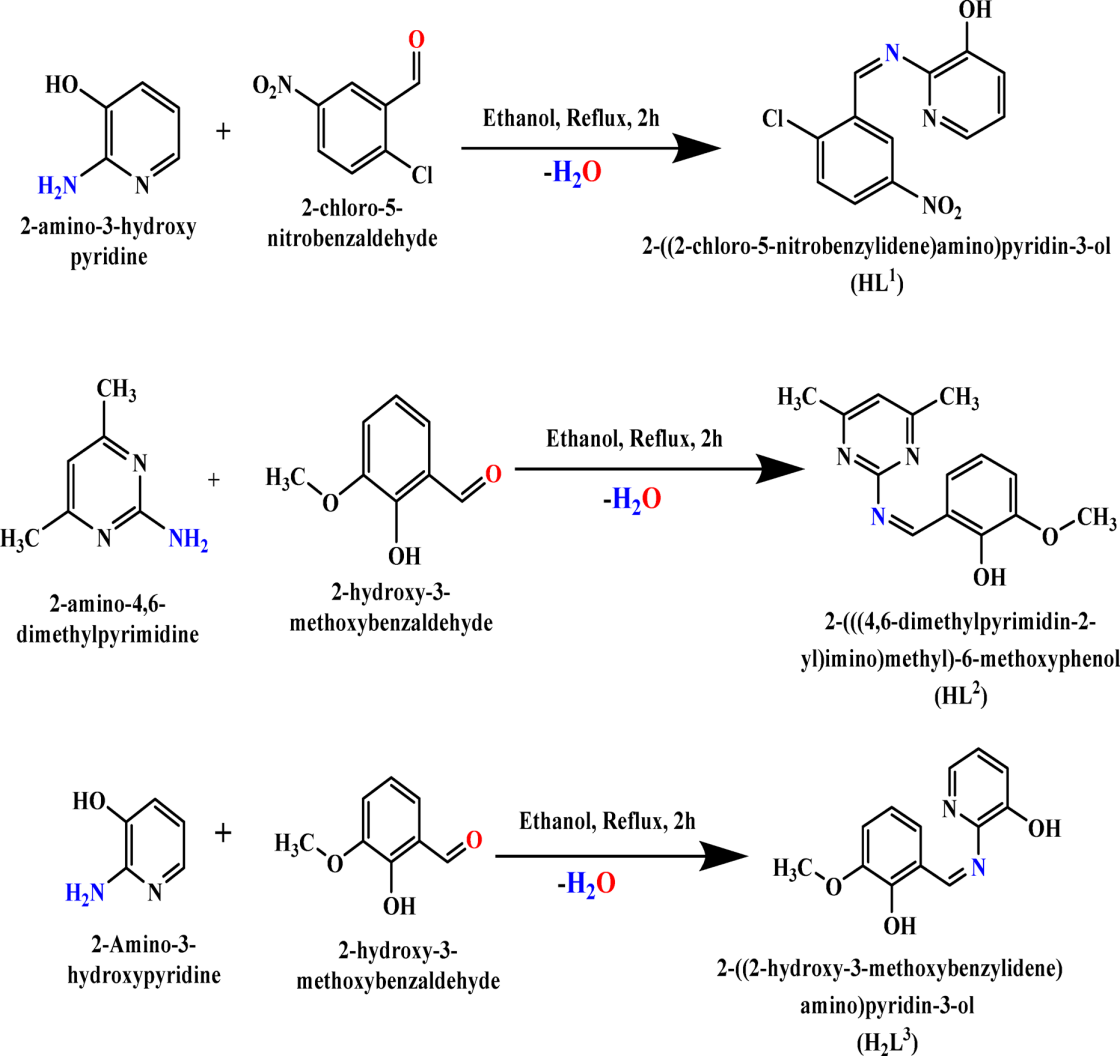
The synthetic pathways for the studied Schiff base ligands (**HL**^**1**^, **HL**^**2**^ and **H**_**2**_**L**^**3**^).

Syntheses of the Schiff bases (**HL**^**1**^ = 2-((2-chloro-5-nitrobenzylidene)amino)pyridin-3-ol, **HL**^**2**^ = 2-(((4,6-dimethylpyrimidin-2-yl)imino)methyl)-6-methoxyphenol and **H**_**2**_**L**^**3**^ = 2-((2-hydroxy-3-methoxybenzylidene)amino)pyridin-3-ol) were carried out by condensing equimolar amounts of either 2-amino-3-hydroxypyridine or 2-amino-4,6-dimethylpyrimidine with either 2-chloro-5-nitrobenzaldehyde or 2-hydroxy-3-methoxybenzaldehyde in absolute ethanol for 2 h. The purity and follow up of the formed ligands were identified using thin layer chromatography (TLC). The resulting imine residues were filtered, recrystallized from hot ethanol, and then kept dry. The synthetic process for the Schiff base ligands is depicted in Fig. 1.

#### Synthesis of the ruthenium(III) complexes

**Fig. 2:**
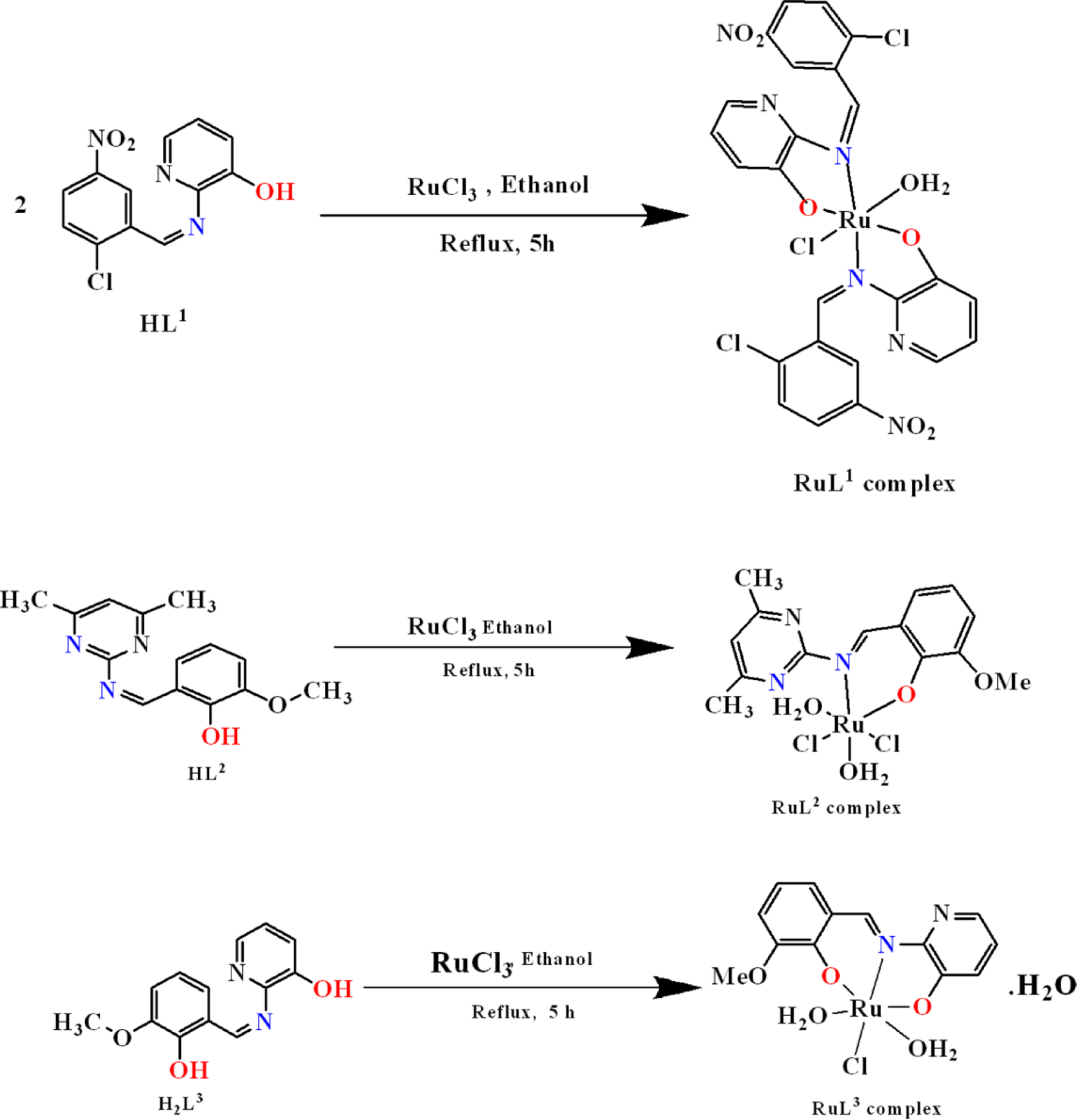
A schematic representation of the synthetic procedures and the proposed structures of ruthenium(III) complexes.

The ruthenium complexes were synthesized by dissolving equimolar amounts (1 mmol) of RuCl_3_ and the respective Schiff base ligand in ethanol, followed by refluxing the mixture under stirring for approximately 5 h. After cooling, the solvent was removed, and the resulting solid was purified by washing with hot petroleum ether and recrystallizing from hot ethanol. Figure 2 visually depicts this synthesis and the proposed molecular structures of the ruthenium complexes.

### DFT analysis

Gaussian 09 W software was utilized to conduct DFT/B3LYP computations for the purpose of determining the energetically optimized geometries of complexes^39^. The LANL2DZ basis set was employed for the ruthenium complexes. Additional experimental details are provided in the Supplementary Information.

### Molecular docking study

The molecular docking analyses were carried out using the Molecular Operating Environment (MOE) program (2014.0901). Two macromolecular biological targets, a penicillin-binding protein 3 from *Escherichia Coli* (PDB ID: 4BJP)^40^ and a B-DNA (PDB ID: 1BNA)^41^ were screened. The PDB files of the targets were obtained from the Research Collaboratory for Structural Bioinformatics (RCSB) database. Preparation of the macromolecular target involved a sequence of actions: removing solvent molecules from the cavity, stabilizing charges, filling missing residues and hydrogens, optimizing the structure, and fixing the potential. The assembly of the docked ligand was drawn in Chemdraw format, inserted into the MOE main page and then carried out structure optimization.

### Biomedical investigations

The antibacterial potency of the ruthenium complexes against *Escherichia coli*, *Serratia marcescens* (Gram-negative), and *Micrococcus luteus* (Gram-positive) was determined using the paper disc diffusion method^42^. The in vitro antitumor screening tests were conducted using three human cancer cell lines: MCF-7 (breast cancer), HCT-116 (colon carcinoma), and HepG2 (liver carcinoma). The cell lines were procured from the VACSERA Tissue Culture Unit, located in Dokki, Giza, Egypt. Detailed experimental procedures are provided in the Supplementary Information.

## Results and discussion

**Fig. 3:**
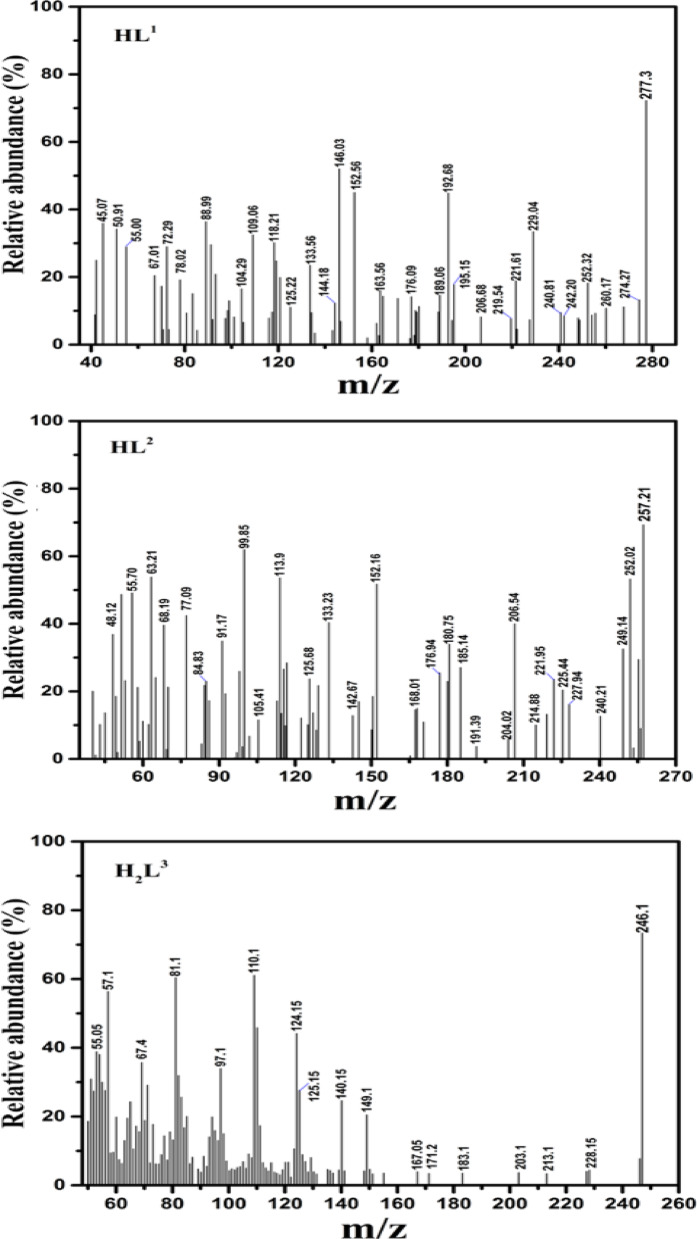
The mass spectra of the Schiff base ligands.

**Fig. 4:**
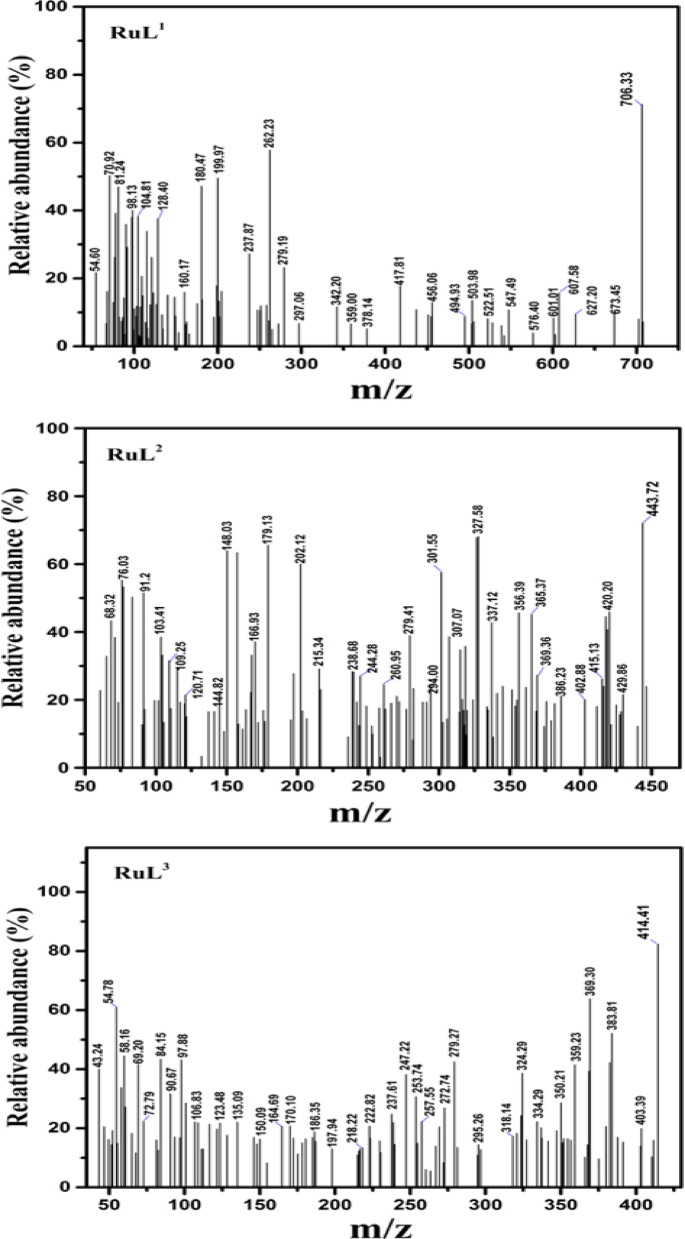
The mass spectra of the developed Ru(III) complexes.

Three mononuclear Ru(III) complexes with bi- and tridentate Schiff base ligands having NO-, NNO- and NOO donor atoms, namely, 2-((2-chloro-5-nitrobenzylidene)amino)pyridin-3-ol (**HL**^**1**^), 2-(((4,6-dimethylpyrimidin-2-yl)imino)methyl)-6-methoxyphenol (**HL**^**2**^), and 2-((2-hydroxy-3-methoxybenzylidene)amino)pyridin-3-ol (**H**_**2**_**L**^**3**^) were synthesized and fully characterized by various analytical and spectroscopic techniques. The Ru-complexes were found to be neutral and have unique intense color and air stability. They were insoluble in most organic solvents except for DMF and DMSO. The molar conductivities (Λm) of 1 × 10^-3^ M DMF solutions of the Ru-complexes ranged between 1.1 and 1.82 Ω^-1^mol^-1^cm^2^ at 25 °C. These low values reflected the non-electrolytic characteristics of the reported complexes^5^. In addition, the magnetic measurements for the Ru(III), d^5^, complexes showed that the values of effective magnetic moments (µ_eff_) were slightly lower than the spin-only value for one unpaired electron (1.45–1.59 BM). The observed magnetic moments were consistent with the postulated octahedral geometry with low-spin electronic configuration. Mass spectrometry analyses were performed for the ligands and complexes to verify the chemical and molecular structures of the new compounds. The mass spectra of the Schiff base ligands displayed prominent ion peaks at 277, 257, and 246, which corresponded to the parent molecular ions of the formulas [C_12_H_8_N_3_O_3_Cl]^+^, [C_14_H_15_N_3_O_2_]^+^, and [C_13_H_12_N_2_O_3_ + 2H]^+^, respectively (Fig. 3). Furthermore, the mass spectrometry results for the ruthenium complexes closely matched the values obtained from elemental analyses, as depicted in Fig. 4. The mass spectrum of **RuL**^**1**^ complex demonstrated a remarkable peak at 706.3, which aligns perfectly with the molecular ion [C_24_H_16_N_6_O_7_Cl_3_Ru-H]^+^. The mass spectrum of the **RuL**^**2**^ complex also exhibited the molecular ion peak at 443.7 corresponding to [C_14_H_16_N_3_O_3_Cl_2_Ru-H_2_]^+^. In addition, the EI-mass spectrum of **RuL**^**3**^ complex showed molecular ion peak at 414.4 corresponding to [C_13_H_16_N_2_O_6_ClRu-H_2_O]^+^. Following and tracking the different fragments in the mass spectra of the reported compounds shed more insight into the structure of them. For example, the mass spectrum of **HL**^**1**^ ligand displayed fragments at m/z = 260 and 242 due to [P-OH]^+^ and [P-HCl]^+^, respectively. In addition, the mass spectrum of **HL**^**2**^ ligand exhibited the fragments [P-C_2_H_6_]^+^ and [P-(OH + C_2_H_6_)]^+^ at m/z = 228 and 214, respectively. For the **H**_**2**_**L**^**3**^ ligand, the mass spectrum showed three fragments at m/z equal to 228, 213 and 149 corresponding to the fragments [P-OH[^+^, [P-2OH[^+^ and [P-(py-OH)]^+^, respectively. On the other hand, the mass spectra of the the three ruthenium complexes displayed fragments that confirm their molecular structures. For instance, the **RuL**^**1**^ complex showed fragments at m/z = 673, 610 and 567 due to the fragments [P-Cl]^+^, [P-(H_2_O + Cl + NO_2_)]^+^ and [P-(H_2_O + 2Cl + NO_2_)]^+^, respectively. The mass spectrum of the **RuL**^**2**^, on the other side, displayed the three fragments [P-H_2_O]^+^, [P-(H_2_O + 2Cl)]^+^ and [P-(H_2_O + Cl + C_2_H_6_)]^+^, at m/z = 429, 357 and 365, respectively. Furthermore, the mass spectrum of the **RuL**^**3**^ complex showed the following fragments: [P-MeOH]^+^ (384), [P-(H_2_O + Cl)]^+^ (360), [P-(2H_2_O + MeOH)]^+^ (348) and [P-(2H_2_O + Cl)]^+^ (343). Microanalytical and relevant physicochemical data for the synthesized Schiff base ligands and their complexes are comprehensively summarized in Table 1.

### FT-IR and ^1^H-NMR investigations

**Fig. 5:**
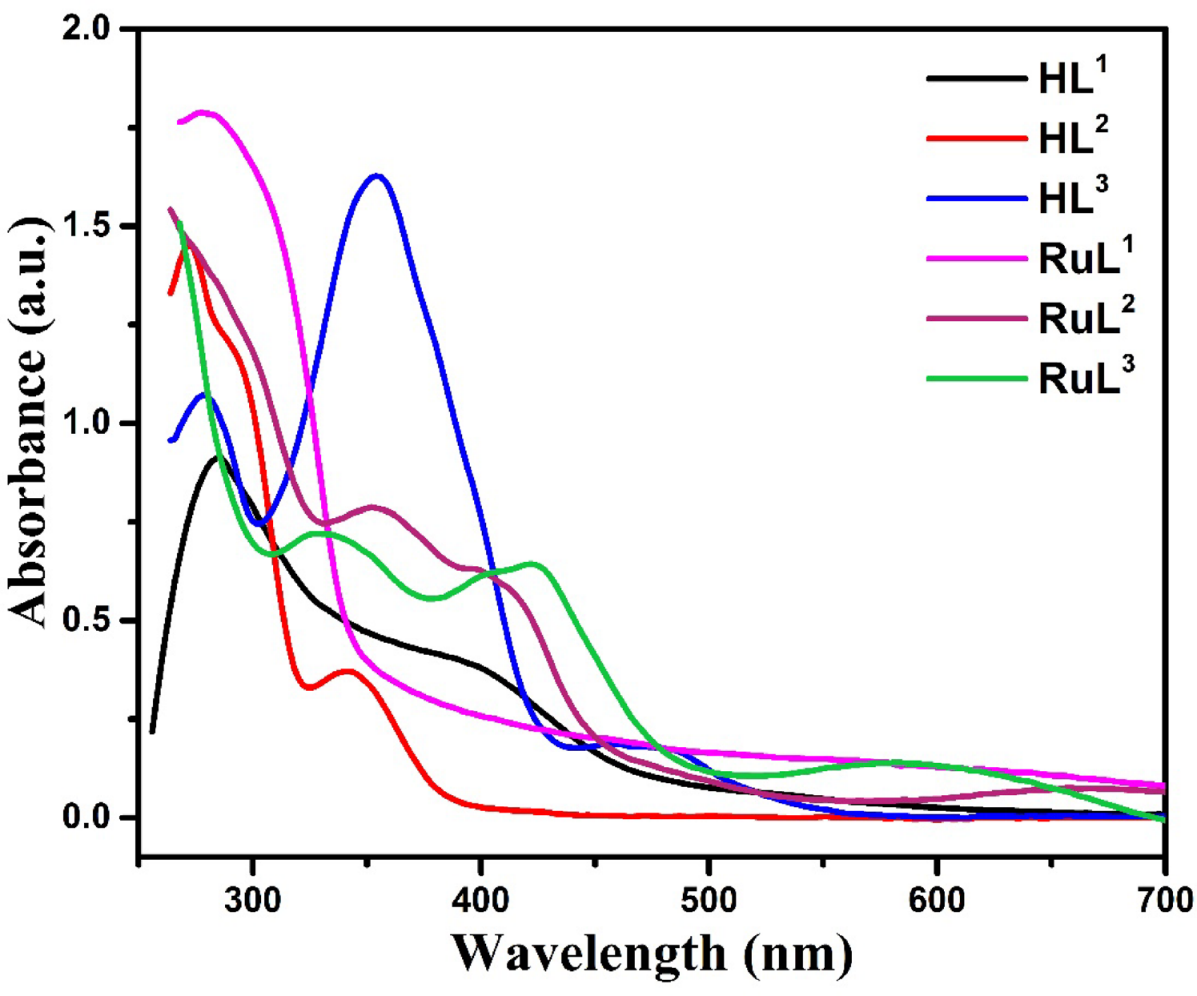
Electronic absorption spectra of ligands and their complexes.

Comparison of the FT-IR spectra of the free Schiff base ligands (Figs. 1 and 2 S) with those of their Ru(III)-complexes (Figs. 4 and 5 S) was conducted to examine the electronic structure and the binding interactions between the ligands and the ruthenium(III) ion in the complex sphere. Table 2 displays the IR data of ligands and their corresponding complexes. Schiff bases can form coordination bonds with metal ions through their azomethine and phenolic functional groups. The free ligands exhibited broad vibrational stretching frequency bands at 3386, 3396 and 3444 cm^− 1^ due to hydroxyl group of the phenolic part of ligands. The observed intensity and spectral range of the υ(OH) band indicated the presence of intramolecular hydrogen bonding between the OH proton and the neighboring azomethine nitrogen^43–45^. It is noteworthy that all of the complexes displayed wide spectral bands within the range of 3374–3419 cm^− 1^, which have been attributed to the stretching vibrations of υ(OH) for the coordinated and/or hydrated water molecules of ruthenium complexes. Furthermore, the infrared spectra of complexes exhibited characteristic in-plane bending of the hydroxyl group, δ(OH), at around 1460 cm^− 1^, which could be attributed to the presence of water molecules associated with the reported complexes^46–48^. The medium-intensity bands detected at 1345, 1384, and 1304 cm^− 1^ of the phenolic C-O stretching of ligands, exhibited shift towards higher wavenumbers, Table 2. These shifts in wavenumbers suggested the involvement of the phenolic oxygen in coordination^49^. In addition, the infrared spectra of the ligands displayed bands at 1613, 1590, and 1619 cm^− 1^ due to the stretching vibrations of the C = N bonds of the azomethine groups. These observed bands underwent frequency shift to a range of 1602–1641 cm^− 1^ because of their coordination with the ruthenium ions via their nitrogen atoms^50^. In addition, the appearance of two weak bands in the spectral range of 507–537 cm^− 1^ and 439–468 cm^− 1^, which can be attributed to the stretching vibrations of the metal-oxygen, υ(M-O), and metal-nitrogen, υ(M-N), bonds, respectively, provided a conclusive proof of the coordination of the azomethine nitrogen and phenolic oxygen atoms to the metal ion^51^.

**Fig. 6:**
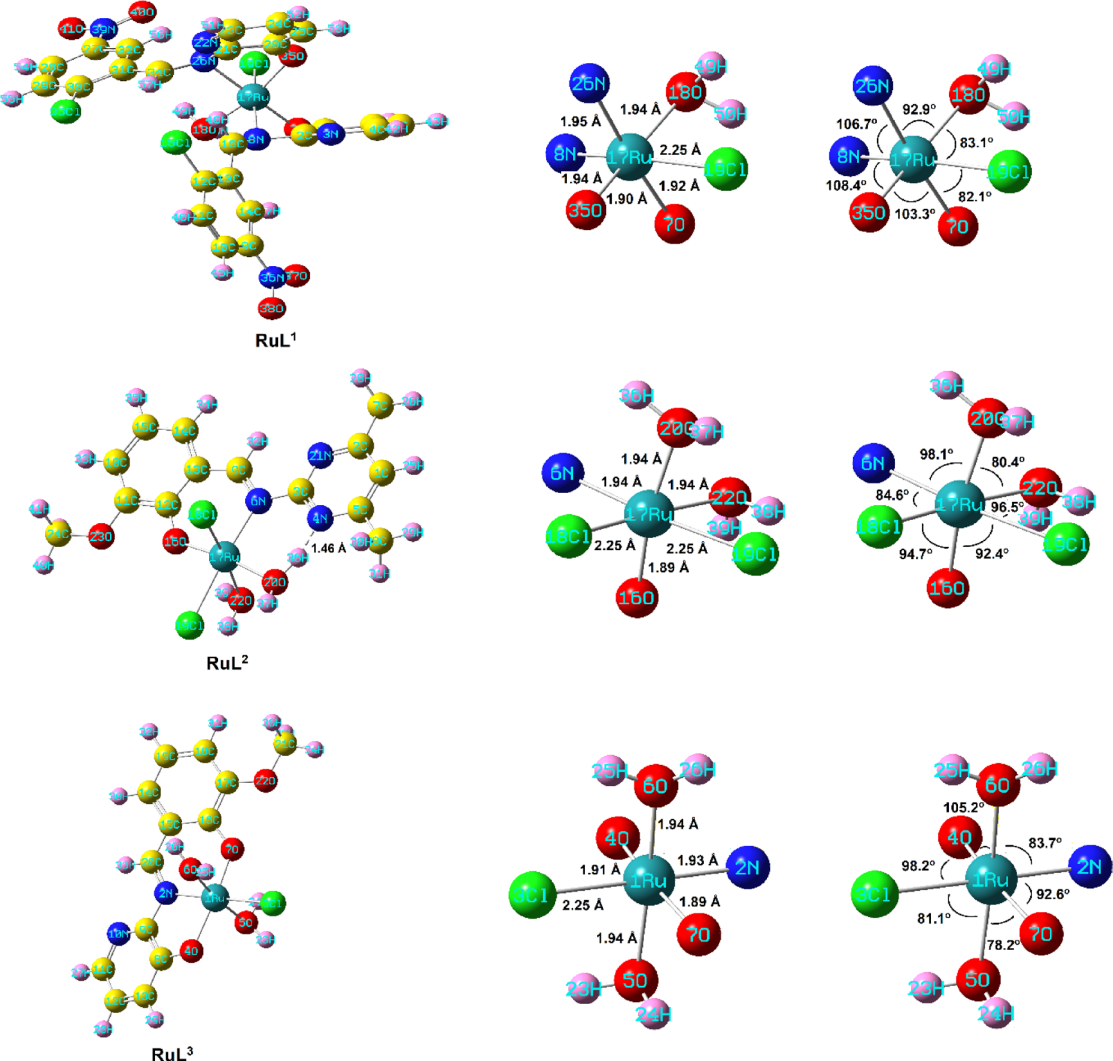
Energy optimized geometries of the studied ruthenium complexes.

**Fig. 7:**
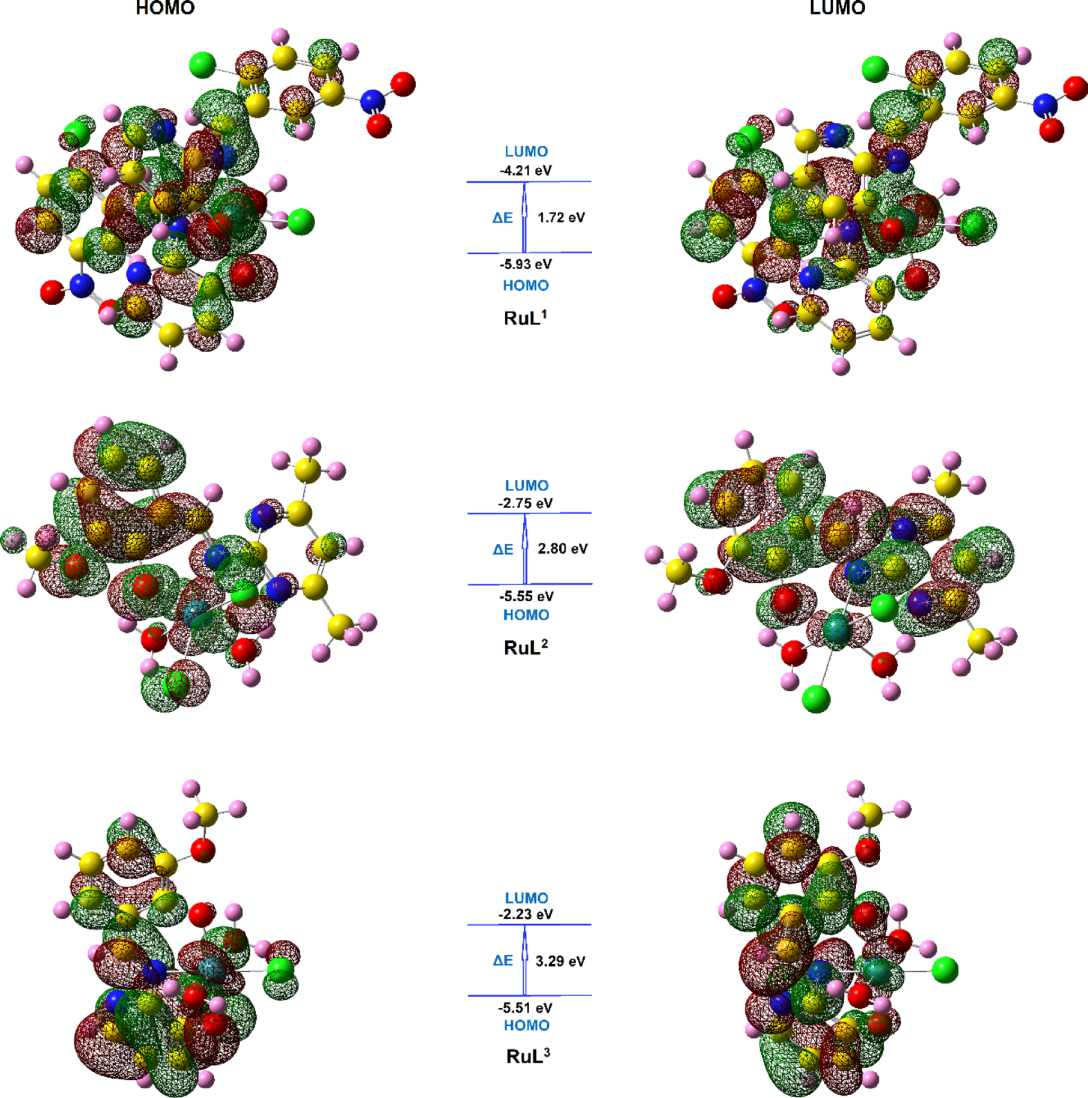
The frontier *HOMO* and *LUMO* orbitals and their energy values.

The ^1^H-NMR spectra of the investigated ligands (Figs. 6 and 7 S) have been studied in DMSO-*d6* at room temperature. Table 3 tabulates the chemical shifts corresponding to the various types of protons observed in the ^1^H-NMR spectrum. The ^1^H-NMR spectra of the **HL**^**1**^, **HL**^**2**^ and **H**_**2**_**L**^**3**^ ligands exhibited singlet peaks at 10.33, 10.26 and 10.25 ppm for the phenolic hydroxyl protons, respectively. In addition, the protons of the azomethine groups displayed singlet peaks at 8.53, and 10.21 and 9.42 ppm. Further, the ^1^H-NMR spectra of the ligands displayed multiple peaks due to the aromatic protons (Table 3). Identification of the (–OCH_3_) moieties of **HL**^**2**^ and **H**_**2**_**L**^**3**^ were also supported from their ^1^H-NMR spectra.

**Table 1:** The microanalyses and physicochemical data for the synthesized compounds.

Compound	Color	Empirical formula	M.wt. (g/mol)	m/z (EI-Mass)	M.*P* (°C)	Found (Calculated)			Λ_m_ (cm^2^ Ω^−1^ mol^− 1^)
						%C	%H	%*N*	
**HL** ^**1**^	Dark brown	C_12_H_8_N_3_O_3_Cl	277.66	277.3	˃300	50.66 (51.86)	2.93 (2.88)	14.79 (15.12)	–
**HL** ^**2**^	Orange	C_14_H_15_N_3_O_2_	257.28	257.2	70	65.06 (65.29)	5.28 (5.83)	13.1 (16.32)	–
**H**_**2**_ **L** ^**3**^	Red	C_13_H_12_N_2_O_3_	244.24	246.1	250	62.5 (63.87)	4.73 (4.91)	11.83 (11.46)	–
**RuL** ^**1**^	Black	C_24_H_16_N_6_O_7_Cl_3_Ru	707.89	706.3	˃300	41.39 (40.68)	3.28 (2.26)	12.51 (11.87)	1.1
**RuL** ^**2**^	Greenish black	C_14_H_18_N_3_O_4_Cl_2_Ru	464.29	443.7	170	37.32 (36.22)	4.09 (3.91)	10.95 (9.05)	1.82
**RuL** ^**3**^	Black	C_13_H_16_N_2_O_6_ClRu	432.79	414.4	˃300	35.88 (36.04)	3.4 (3.7)	7.33 (6.47)	1.63

**Table 2:** FT-IR spectral data for the ligands and their ruthenium complexes.

Compound*	υ(OH)_L_	υ(OH)_W_	δ(OH)	υ(C = *N*)	υ(C-O)_phenolic_	υ(M-O)	υ(M-*N*)
**HL** ^**1**^	3386	–	1490	1613	1345	-	–
**HL** ^**2**^	3396	–	1460	1590	1384	-	–
**H**_**2**_ **L** ^**3**^	3444	–	1464	1619	1304	-	–
**RuL** ^**1**^	–	3375	1495	1614	1346	526	467
**RuL** ^**2**^	–	3419	1478	1602	1367	507	439
**RuL** ^**3**^	–	3375	1462	1641	1318.	536	446

* L ligand, *W* water.

**Table 3:** The ^1^H-NMR spectral data (δ, ppm) of the schiff base ligands.

Compound	(-OH)_phenolic_	HC = *N*	-OCH_3_	Aromatic protons
**HL** ^**1**^	10.33 (s)	8.53 (s)	−	6.42–7.99 (m)
**HL** ^**2**^	10.26 (s)	10.21(s)	3.84 (s)	6.29–7.25(m)
**H**_**2**_ **L** ^**3**^	10.25 (s)	9.42 (s)	3.81 (s)	6.39–7.41 (m)

### Electronic absorption studies

The electronic absorption spectra of the reported compounds were performed in DMF at ambient temperature, Table 4; Fig. 5. The absorption spectra of the ligands displayed two high intensity and were assigned to π–π* and n–π* transitions due to electrons on benzene rings and the C = N chromophore^52^. These bands were shifted in the spectra of complexes due to coordination of nitrogen pair of electrons to the metal and hence the bonding of azomethine to the metal ions. In addition, the complexes exhibited a weak broad band in the 400–500 nm range due to ligand-to-metal charge transfer (LMCT) transitions^53,54^.

**Table 4:** Electronic absorption transitions data for the ligands and their ruthenium complexes.

Compound	UV-vis (nm, DMF)	Assignments
**HL** ^**1**^	286, 392	π-π*, n-π*
**HL** ^**2**^	272, 342	π-π*, n-π*
**H**_**2**_ **L** ^**3**^	278, 354	π-π*, n-π*
**[Ru(L** ^**1**^ **)**_**2**_ **(H**_**2**_ **O)(Cl)]**	280, 454	π-π*, CT
**[RuL** ^**2**^ **(H**_**2**_ **O)(Cl)**_**2**_ **]**	264, 354, 410	π-π*, n-π*, CT
**[RuL** ^**3**^ **(H**_**2**_ **O)**_**2**_ **(Cl)].H**_**2**_ **O**	266, 332, 422	π-π*, n-π*, CT

### Thermal analysis

The thermal analysis approach is widely regarded as a highly effective method for determining the quantity and nature of water molecules associated with metal complexes. This is achieved by evaluating the percentage of mass loss through the heating process^55^. Therefore, the thermogravimetric analysis of the three ruthenium complexes was carried out in air, reaching temperatures up to 1000 °C with a heating rate of 10 °C per minute, Figs. (10–12 S). The results obtained demonstrated that the complexes under investigation exhibited significant thermal stability. The decomposition processes of these complexes occurred in two or three distinct steps, involving the partial detachment of the hydrated and coordinated water along with the organic ligands’ moieties (Table 5). Ultimately, the decomposition ended with the formation of metallic oxide and carbide residues.

The breakdown action of **RuL**^**1**^ complex comprised two distinct decomposition stages. The initial stage took place within the temperature range of 36–418 °C and was associated with the release of coordinated H_2_O and C_3_H_9_NOCl fragments. During the ensuing second stage, which occurred within the temperature range of 419–755 °C, the liberation of C_5_H_5_N_5_O fragments was observed, resulting in the formation of metallic ruthenium residue. On the other hand, three distinct phases were involved in the decomposition of the **RuL**^**2**^ complex. The first phase involved the release of coordinated water (H_2_O) and C_2_H_2_N_2_ fragments, and took place between 36 and 232 °C. The second stage was defined by the release of C_6_H_10_N moieties and occurred within the 233–388 °C range. The third instance of mass loss occurring within the temperature range of 389 to 589 °C was attributed to the liberation of 2HCl, leading to the formation of metallic residue. Ultimately, the **RuL**^**3**^ complex experienced disintegration via a sequence of three discernible decomposition phases. The first phase, occurring between temperatures of 33–140 °C, involved the release of a water molecule in its hydrated form. The second phase (141–390 °C) was characterized by the release of CH_2_NCl species and two coordinated H_2_O. The third decomposition stage that took place in the temperature range of 391 to 550 °C was ascribed to the dissociation of C_7_H_6_N, leading to the formation of metallic ruthenium residue.

**Table 5:** Thermogravimetric data of the studied ruthenium complexes (**1–3**).

Compound	Empirical formula	Temp. range (°C)	Mass loss (%)		Assignment
			Found	Calc.	
**RuL** ^**1**^	C_24_H_16_N_6_O_7_Cl_3_Ru	36–418	31.49	32.71	H_2_O_(coordinated)_ + C_3_H_9_NO_3_Cl_3_
		419–755	20.51	21.33	C_5_H_5_N_5_O
**RuL** ^**2**^	C_14_H_18_N_3_O_4_Cl_2_Ru	36–232	18.34	19.38	2H_2_O_(coordinated)_ + C_2_H_2_N_2_
		233–388	21.19	20.68	C_6_H_10_N
		389–589	16.46	15.72	2HCl
**RuL** ^**3**^	C_13_H_16_N_2_O_6_ClRu	33–140	3.15	4.16	H_2_O_(hydrated)_
		141–390	21.79	22.99	CH_2_NCl + 2 H_2_O_(coordinated)_
		391–550	25.45	24.03	C_7_H_6_N

### Molecular orbital calculations

The energy optimized geometry, structural parameters, and the global reactivity parameters of the three ruthenium complexes were performed using DFT/B3LYP method. The energetically stable structures of the complexes were determined by focusing on the ruthenium core and the spatial arrangement of coordinated HC = N, OH, and water molecules. The energetically optimized geometries of the ruthenium complexes, **RuL**^**1**^, **RuL**^**2**^ and **RuL**^**3**^, were investigated and the calculated energy minimized structures, numbering systems, lengths of bonds, and angle values around the metal are shown in Fig. 6. All the complexes illustrated energetically stable patterns with distorted octahedral arrangement and minimization energy = 247.22, 127.34 and 184.95 kcal/mol for **RuL**^**1**^, **RuL**^**2**^ and **RuL**^**3**^, respectively. The distortion of the octahedral structures is clearly noticed from the deviation of the bond angles and bond lengths from the regular octahedron arrangement. It is worth mentioning that the N-Ru-N, N-Ru-O and O-Ru-O angles in the three complexes are wider than the other bond angles in the coordination core (Fig. 6). This could be probably due to the repulsive forces between the electronegative donor atoms. Interestingly, the **RuL**^**2**^ complex illustrated intramolecular hydrogen bond between one of the pyrazine nitrogen and proton of the adjacent coordinated water (H…*N* = 1.46 Å. It is noteworthy that the remaining bond lengths and angles exhibited values consistent with those typically observed in related structures^45,56,57^.

The highest occupied molecular orbital (*HOMO*) and the lowest unoccupied molecular orbital (*LUMO*) in the molecular orbital diagram of a molecule are very useful expressions for the optical, electric properties and charge transfer (CT) transitions of that molecule^44,58^. Figure 7 represents the *HOMO* and *LUMO* orbitals and energies of the investigated ruthenium complexes as well as the energy gap (*ΔE*) values. The reactivity descriptors of the complexes, such as *E*_*HOMO*_, *E*_*LUMO*_, energy gap (*ΔE*), chemical hardness (*η*), chemical potential (*V*), electron affinity (*EA*), ionization potential (*IP*), electronegativity (*χ*), electrophilicity index (*ω*) and chemical softness (*S*) were determined by the DFT calculation and are tabulated in Table 6. The donation characteristics within the molecule as represented by *E*_*HOMO*_ values are almost equal for the three complexes, while the accepting properties reflected by the *E*_*LUMO*_ values are diverse. The **RuL**^**1**^ complex showed the highest negative *E*_*LUMO*_ value and consequently the lowest energy gap (*ΔE* = 1.72 eV), which might explore its increased degree of reactivity and would have facile charge transfer and polarization within the molecule. Variations in electronegativity (χ) are related to differences in electrostatic chemical potentials. The values of *χ* for the investigated complexes have the order **RuL**^**1**^ > **RuL**^**2**^ > **RuL**^**3**^. The capacity for charge transfer within these derivatives is reflected in their chemical hardness (η) parameters. Notably, the order of chemical hardness is inversely related to the order of electronegativity. Furthermore, the electrophilicity index (*ω*) of the complexes has a reverse order to their chemical softness (*S*) values that reflected to their biological properties.

**Table 6:** The global chemical reactivity descriptors for the ruthenium complexes.

Reactivity descriptor	RuL^1^	RuL^2^	RuL^3^
Total energy (au)	−1917.40	−1133.26	−1098.23
*DM* (Debye)	9.37	11.26	5.32
*HOMO* (eV)	−5.93	−5.55	−5.51
*LUMO* (eV)	−4.21	−2.75	−2.23
*ΔE* (eV)	1.72	2.80	3.29
*Χ* (eV)	5.07	4.15	3.87
*V* (eV)	−5.07	−4.15	−3.87
*EA* (eV)	4.21	2.75	2.23
*IP* (eV)	5.93	5.55	5.51
*η* (eV)	0.86	1.40	1.64
*S* (eV)	0.43	0.70	0.82
*ω* (eV)	14.96	6.14	4.56

### Biological studies

#### In vitro antimicrobial activities of the reported complexes

The biological functions of metallic complexes are determined by a combination of several factors. These include the chelate effect exhibited by Schiff base ligands, the specific characteristics of the donor atoms involved, the overall charge carried by the complex, the identity of the metal ion, the nature of the counter ions that neutralize the complex, and the geometric arrangement of the complex^59,60^. The process of chelation leads to a decrease in the polarity of the metal ion. This reduction occurs through a mechanism involving the partial sharing of the positive charge of the metal ion with the donor groups of the ligand. Additionally, the delocalization of π-electrons across the entire chelate ring system may further contribute to this reduction in polarity^61,62^. Hence, these constituents enhance the lipophilic nature of the metal atom core, thereby augmenting the hydrophobicity and lipo-solubility of the compound. This enables the compound to effectively interact with or traverse the lipid bilayers of the microorganism membrane. Consequently, the studied antimicrobial activity of the chemicals is enhanced due to the increased rate of absorption or entrance. Because of the ability of numerous pathogenic microbes to acquire antibiotic resistance through biochemical and morphological changes, we deemed it was necessary to investigate the antimicrobial properties of the reported ruthenium complexes as novel potential antibiotic agents.

**Fig. 8:**
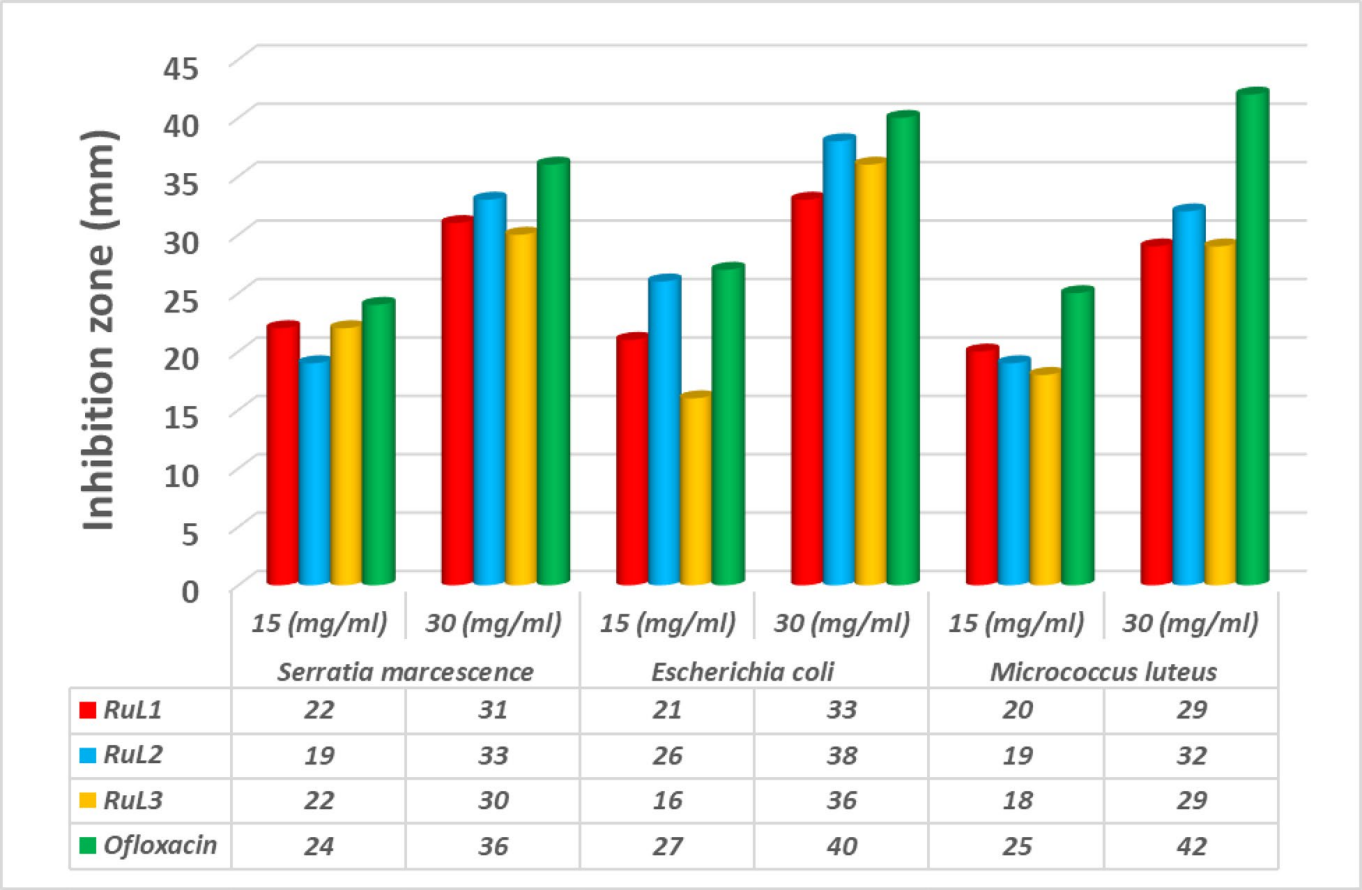
The inhibitory potency of the ruthenium complexes against the tested bacteria.

**Fig. 9:**
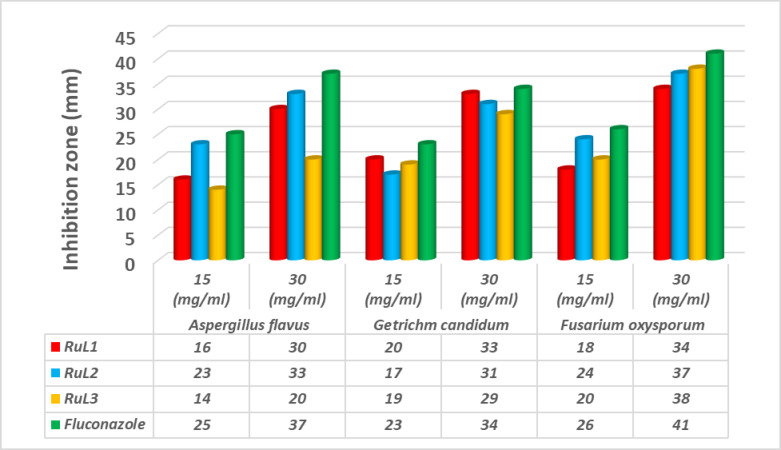
The inhibitory potency of the complexes against the tested fungi.

The ruthenium complexes were evaluated for their inhibitory impacts on the growth of the G^+^ bacteria *Micrococcus luteus* and the two G^−^ bacteria *Escherichia coli* and *Serratia marcescens* as well as the three fungi *Aspergillus flavus*,* Geotrichum candidum* and *Fusarium oxysporum*. The antibacterial and antifungal activities of the complexes are represented graphically in Figs. 8 and 9. The antimicrobial efficacy of the compounds were evaluated using two different doses (15 and 30 mg/mL). The inhibitory potencies were remarkably increased by increasing the concentrations of the compounds investigated. The results indicated that the Ru complexes exhibited rather high activities against the tested bacterial or fungal strains, and they were comparable to those of the standards. The order of the bactericidal activity against the bacteria varied upon changing the concentration of the tested compound. Thus, at the low concentration (15 mg/mL), the activity order against the *M. luteus* bacteria was **RuL**^**1**^ > **RuL**^**2**^ > **RuL**^**3**^, for the *E. Coli* bacteria, the order of activity was **RuL**^**2**^ > **RuL**^**1**^ > **RuL**^**3**^, whereas the inhibition effect of the compounds on the *S. marcescens* bacteria was almost equal. On the other hand, at a concentration of 30 mg/mL, the tested compounds demonstrated good antifungal activity, comparatively slightly less than the Fluconazole standard. The fungicidal activity against *A. flavus* followed the order: **RuL**^**2**^ > **RuL**^**1**^ > **RuL**^**3**^, whereas versus *G. candidum*, the order of activity was **RuL**^**1**^ > **RuL**^**2**^ > **RuL**^**3**^. For *F. oxysporum*, the fungicidal activity of the complexes had the order: **RuL**^**2**^ ≈ **RuL**^**3**^ > **RuL**^**1**^. The mechanism of efficacy of the tested compounds on the tested microbes could be due to the permeability of the complexes through the microbial cells or alterations in microbial ribosomes^63^. Furthermore, the polarity of the metal was diminished through chelation due to the involvement of the partial exchange of positively charged metal ions with the donor groups found in the overlapping ligand orbitals. Therefore, it could facilitate the delocalization of the π-electrons across the entire chelate ring, and hence enhance the ability of the complex to penetrate lipid membranes^64^. Consequently, the cell respiration might be disrupted by the complex causing the prevention of the protein synthesis, which limited the organism growth^65,66^.

#### In vitro cytotoxicity studies

**Fig. 10:**
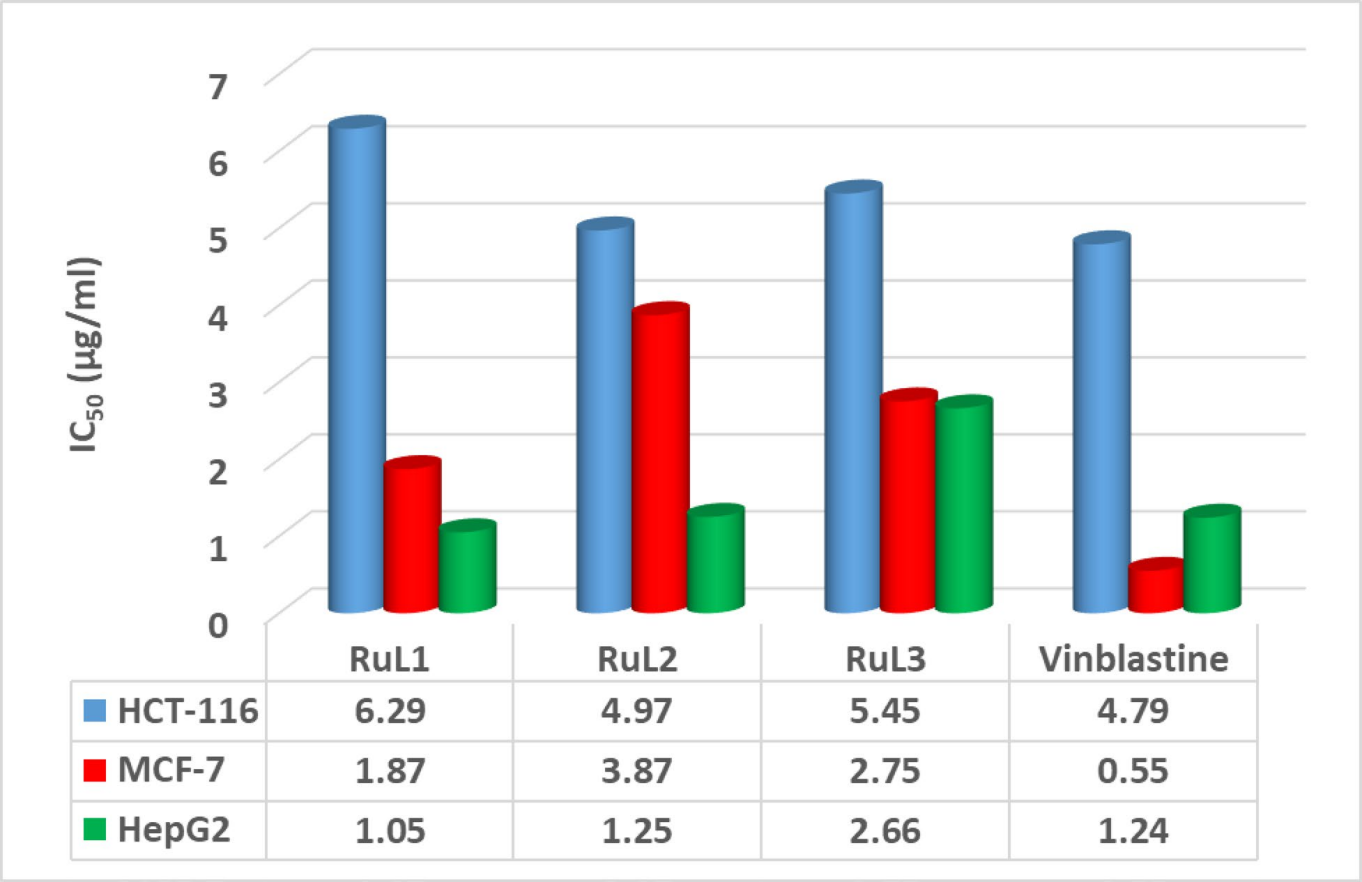
The cytotoxic activity (IC_50_) of the investigated complexes against the HCT-116, MCF-7, and HEPG2 cell lines.

The in vitro cell viability of the HCT-116, MCF-7, and HEPG2 cancer cell lines was assessed by the reported ruthenium complexes. The chemotherapy medication Vinblastine was employed as a reference drug. The assay revealed that the examined complexes have high cytotoxic activities towards the tested cell lines. Figure 10 illustrated the IC_50_ values (concentration of a drug or inhibitor required to inhibit a biological process by 50%) for the complexes. According to the findings, the **RuL**^**2**^ complex showed the lowest IC_50_ value (4.97 µg/mL) versus the HCT-116 cell line, which is very close to that of the reference drug. The order of cytotoxic activity was: **RuL**^**2**^ > **RuL**^**3**^ > **RuL**^**1**^. On the other hand, the **RuL**^**1**^ and **RuL**^**2**^ complexes demonstrated the most pronounced cytotoxic effects against the HEPG2 cell line, which were also close to the value of Vinblastine reference drug. Several biological factors could explain the enhanced response of HepG2 cells to **RuL**^**1**^ **and RuL**^**2**^. HepG2 cells may express higher levels of liver-specific receptors or transporters, such as organic anion-transporting polypeptides (OATPs), which could facilitate the increased uptake of the ruthenium complexes^67^. Additionally, liver metabolism may contribute to higher intracellular accumulation, as HepG2 cells possess elevated levels of metabolic enzymes involved in drug uptake and transformation. Differences in cellular pathways, such as redox potential, could also favor the activation of these complexes, enhancing their cytotoxic effects^68^. Furthermore, the introduction of chloro and nitro groups could increase the lipophilicity of the complexes, promoting better membrane penetration and more efficient cellular uptake, while also influencing their interaction with key cellular receptors and pathways, such as redox regulation and DNA damage recognition, thereby contributing to selective toxicity in liver cancer cells^69^. Many investigations of ruthenium complexes, especially those of bioactive pyrimidine ligands, explored their privileged activity against HEPG2 cell line. A specific ruthenium(III) Schiff base complex, containing 2-chloro-5-nitrophenyl and 4,6-dimethylpyrimidinyl groups, has demonstrated potent anticancer activity against the HEPG2 cell line, with an IC50 of 29 µM. This complex triggers cell death (apoptosis) and halts cell division in the S phase, suggesting its potential as a therapeutic drug, as supported by biological and molecular docking studies^30^. These findings are particularly encouraging when compared to the cytotoxic activity of the standard medications. The sequence of activities against HEPG2 cell line was determined to be **RuL**^**1**^ **> RuL**^**2**^ **> RuL**^**3**^. The IC50 data (µg/ml) for the studied complexes, relative to the Vinblastine standard, are illustrated in Fig. 10.

### Molecular docking

**Fig. 11:**
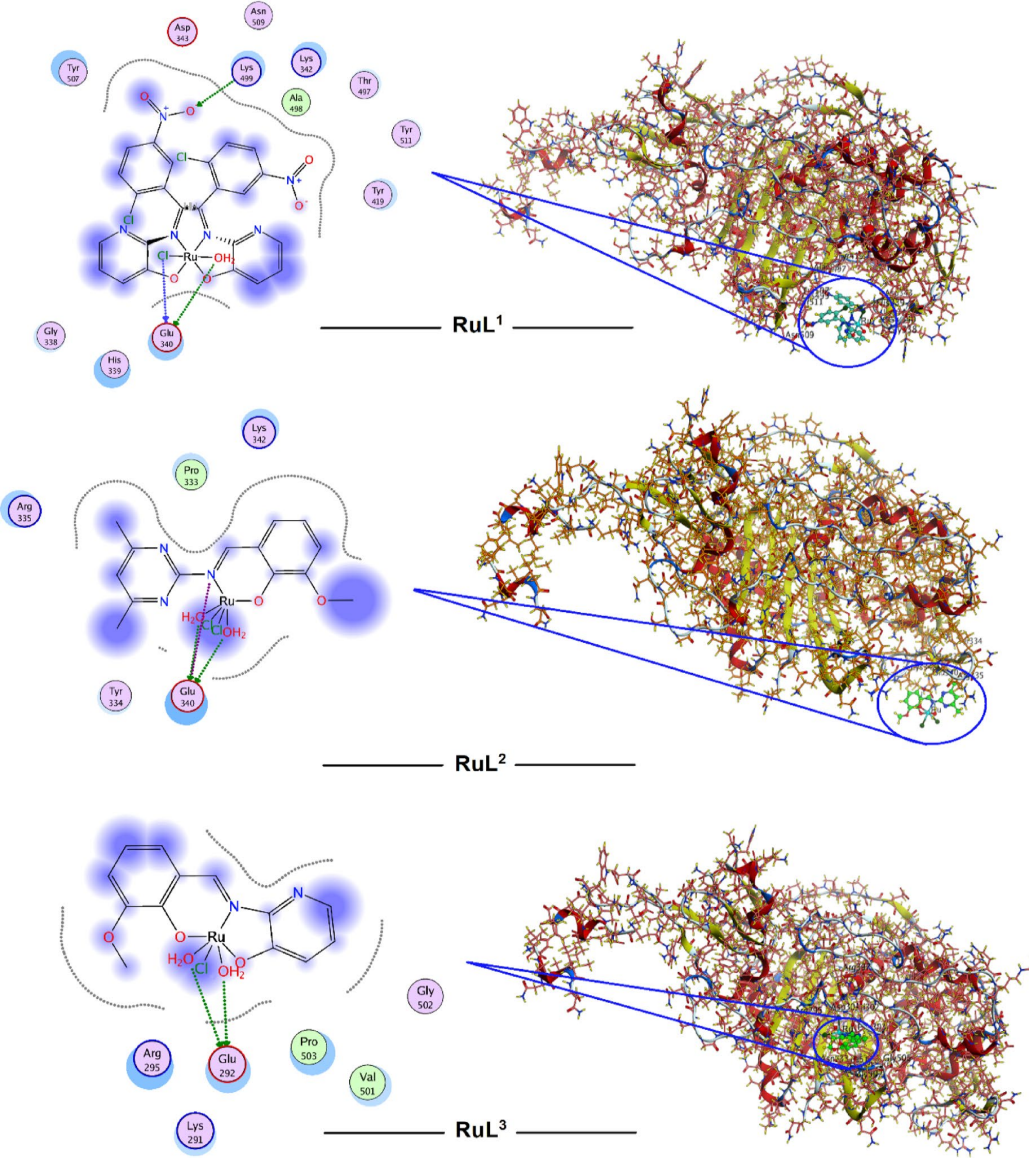
*E. Coli* (PDB ID: 4BJP) docking interactions with investigated Ru complexes.

**Fig. 12:**
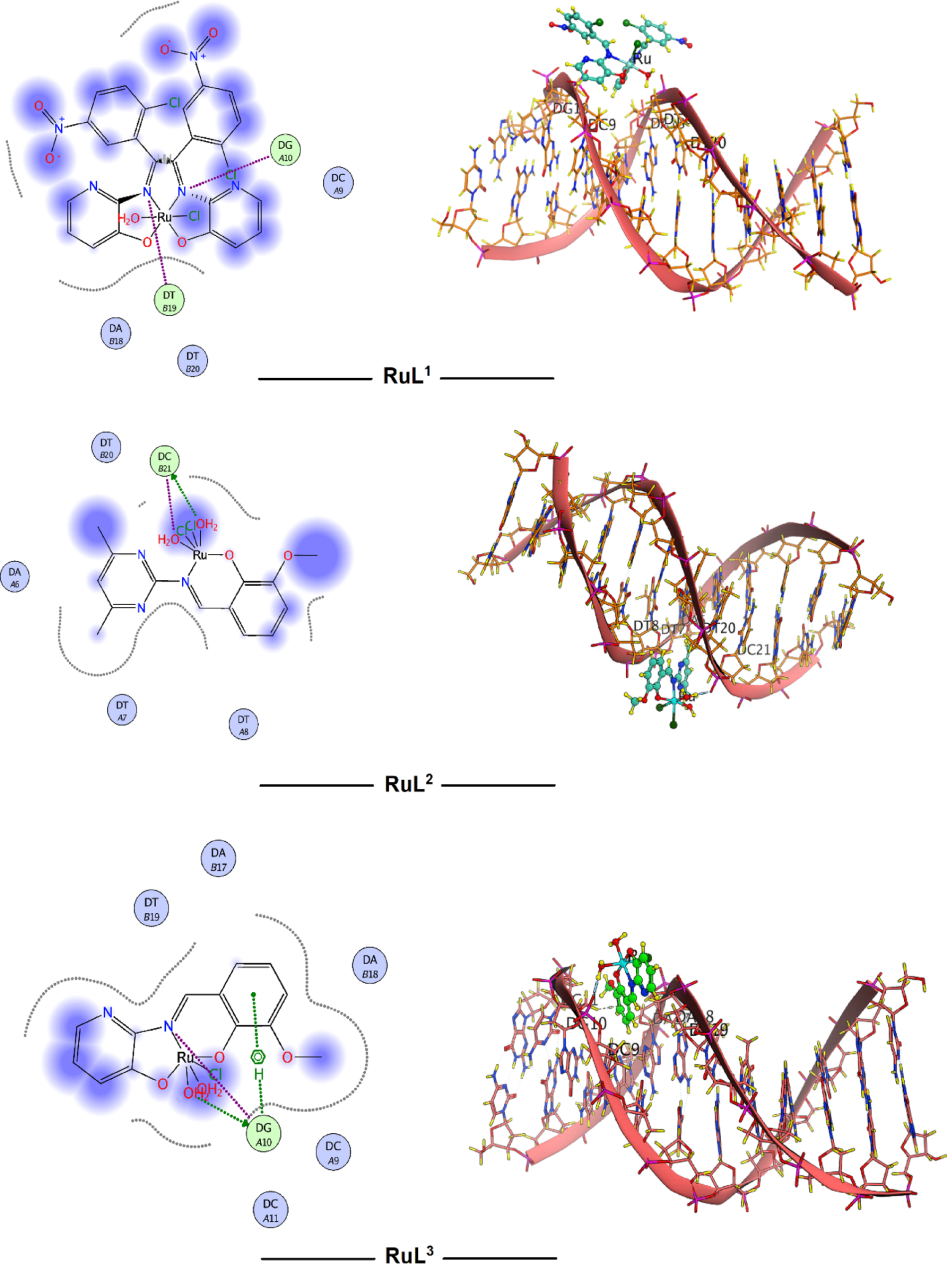
B-DNA (PDB ID: 1BNA) docking interactions with investigated Ru complexes.

In silico molecular docking is a widely used computational technique to simulate the binding interactions between small, synthesized molecules and biological macromolecules (receptors) such as proteins or DNA. These molecular interactions play crucial roles in numerous biological processes^70,71^. Understanding the structures and binding sites of biologically active receptors is crucial for elucidating the diverse binding modes and affinities between interacting molecules. Bioactive Schiff base derivatives and their transition metal complexes have emerged as promising pharmacophores with significant applications in medicine and pharmaceutical chemistry^72^. Extensive molecular docking studies were conducted to investigate the interactions of the bioactive Schiff base metal complexes with a wide range of macromolecular targets^71^. Therefore, the reported ruthenium complexes were subjected to molecular docking determination to realize the types of compound-target interactions and to recognize the potential binding modes and energy. The methodology of molecular docking was validated to guarantee that the procedures and input parameters were reliable and generate accurate prediction for the guest-host interactions. So, the validation operation was constructed on many factors like RMSD (root mean square deviation) value, scoring function values and the amounts and types of binding interactions. The RMSD values are considered the important key parameters for concluding the accuracy of the docking procedure. The RMSD value estimates the average space between the atomic positions of the docked guest and its original conformation. The method is considered optimally accepted with high precision when the RMSD value is less than 2 Å. However, the method is still acceptable and reasonably reliable if the RMSD value is between 2 and 3 Å^73^. Penicillin-binding protein 3 from *Escherichia coli* and B-DNA were selected as macromolecular biological targets for molecular docking studies. This selection aims to correlate the computational data obtained from docking analysis with the experimental results from antibacterial and cytotoxicity assays. Values of binding score (S), hydrophobic, and different H-bonding interactions were checked to evaluate the different conformations of the docked species with the biological targets. The high negative docking scores presented in Table 7 indicate strong binding affinities of the ruthenium complexes to the investigated targets, suggesting their potential efficacy. Figures 11 and 12 visually depict the various docking interactions, while Table 7 provides a comprehensive summary of the calculated docking results, including score function values, root-mean-square deviation (RMSD) values, and the types and energies of bonding interactions.

The amino acid fragments lysine (Lys), glutamic acid (Glu), glycine (Gly), aspartic Acid (Asp), threonine (Thr), asparagine (Asn), valine (Val), arginine (Arg), tyrosine (Tyr), alanine (Ala), and proline (Pro) were the most interacted residues of the target 4BJP (*E coli*) with the docked ruthenium complexes (Fig. 11). The interactions varied between polar-sidechain acceptors (Tyr, Asn, Thr and Gly), acidic-sidechain donors (Glu and Asp), basic-backbone acceptors (Lys and Arg), and backbone donors (Ala, Pro and Val) along with ionic interactions (Table 7). All the three ruthenium complexes displayed almost the same binding affinity towards 4BJP target (S = ≈ -3.8 kcal/mole). Such behavior is correlated with the experimental findings as the complexes exhibited comparable activity towards *E coli* bacteria especially at 30 mg/ml concentration (Fig. 8).

The three ruthenium complexes interacted with DG, DT, DC, and DA nucleotide residues of the DNA (1BNA) receptor via either backbone donor or ionic-metal complex interactions (Table 7; Fig. 12). According to the score function values, the molecular binding abilities were ordered as: **RuL**^**3**^ > **RuL**^**2**^ > **RuL**^**1**^. While the observed order of cytotoxic effects against the tested cell lines did not perfectly align with the order suggested by the docking studies, the combined results from both experimental and computational analyses strongly suggest that the reported ruthenium complexes possess significant potential as bioactive agents and warrant further investigation as potential drug candidates.

**Table 7:** The molecular Docking interaction data for the ruthenium complexes.

Compound		RMSD value^*^ (Å)	S (kcal/mol)	Ligand-receptor	Interaction type	Interaction distance (Å)	E (kcal/mol)
**RuL** ^**1**^	**1BNA**	1.71	−3.33	N11-OP1 (DG 10 A) N38-OP1 (DT 19B)	Ionic Ionic	3.60 3.93	−1.5 −0.7
	**4BJP**	0.99	−3.86	O25-OE1 (GLU 340 A) CL28-O (GLU 340 A) O54-NZ (LYS 499 A) O25-OE1 (GLU 340 A)	H-donor H-donor H-acceptor Ionic	2.86 3.01 3.19 2.86	−8.7 −0.4 −2.5 −5.5
**RuL** ^**2**^	**1BNA**	2.64	−4.30	O35-OP1 (DC 21B) O31-OP1 (DC 21B) O35-OP1 (DC 21B)	H-donor Ionic Ionic	2.72 3.03 2.72	−22.5 −4.3 −6.7
	**4BJP**	2.06	−3.85	O31-OE2 (GLU 340 A) O35-OE1 (GLU 340 A) O35-OE2 (GLU 340 A) N7-OE2 (GLU 340 A) O31-OE2 (GLU 340 A) O35-OE1 (GLU 340 A) O35-OE2 (GLU 340 A)	H-donor H-donor H-donor Ionic Ionic Ionic Ionic	2.71 3.21 2.91 3.10 2.71 3.21 2.91	−25.2 −8.7 −1.7 −3.8 −6.7 −3.2 −5.1
**RuL** ^**3**^	**1BNA**	1.67	−5.18	O8-OP1 (DG 10 A) N2-OP1 (DG 10 A) O8-OP1 (DG 10 A) 6-ring-C5’ (DG 10 A)	H-donor Ionic Ionic π-H	2.67 3.50 2.67 3.48	−19.8 −1.9 −7.1 −0.6
	**4BJP**	1.05	−3.84	O5-OE1 (GLU 292 A) O8-OE1 (GLU 292 A) O5-OE1 (GLU 292 A) O8-OE1 (GLU 292 A) O8-OE2 (GLU 292 A)	H-donor H-donor Ionic Ionic Ionic	2.92 2.91 2.92 2.91 3.81	−17.2 −21.1 −5.1 −5.1 −0.9

## Conclusions

Novel mononuclear Ru(III) complexes with three diverse Schiff base ligands were successfully synthesized, exhibiting notable correlations between their structure and biological activity. Analysis of the molecular geometries and global reactivity parameters of the complexes by DFT method exhibited their unique structural arrangements and reactivity. Notably, the ruthenium complexes displayed antibacterial and antifungal activities, on par with established standard compounds. The order of bactericidal activity varied depending on the concentration of the tested compounds, highlighting their potential for selective and concentration-dependent antimicrobial applications. The biological and molecular docking investigations revealed their potential for use in various applications, including antibiotic and anticancer treatments. The integration of theoretical and experimental findings provided deeper insights into the possible applications of these complexes as bioactive agents, indicating their potential as therapeutic drugs. So, further in vivo studies are eagerly required to explore the potent capabilities of these complexes to be used as effective reagents in the pharmacology fields. Future research endeavors encompass the synthesis and characterization of structurally analogous Ru(III)-Schiff base complexes featuring modified ligands with the objective of assessing potential enhancements in their biological activities. Additionally, exploring structural modifications could enhance selectivity and potency, paving the way for the development of targeted metal-based drugs.

## Electronic supplementary material

Below is the link to the electronic supplementary material.

Supplementary Material 1

## Data Availability

All data generated or analyzed during this study are included in this published article and its supplementary information files.
