# Supplementary material for: Structural and biomedical investigations of novel ruthenium schiff base complexes
Source: Sci Rep. 2025 May 27;15:18546. doi: 10.1038/s41598-025-03147-9 (PMC12117082; doi:10.1038/s41598-025-03147-9)
Supplement: Supplementary file 1 — Supplementary Material 1 [file 41598_2025_3147_MOESM1_ESM.docx]

**Supplementary Information**

**Structural and biomedical investigations of novel ruthenium Schiff base complexes**

Ramadan M. Ramadan, Hadeel H. El-Shalakany, Mostafa A. Sayed*

*Chemistry Department, Faculty of Science, Ain Shams University, 11566, Cairo, Egypt*

**Corresponding Author: Mostafa A. Sayed (mostafa_abdellah@sci.asu.edu.eg)*

***DFT analysis***

Gaussian 09W software was utilized to conduct DFT/B3LYP computations for the purpose of determining the energetically optimized geometries of complexes **[1]**. The LANL2DZ basis set was employed for the ruthenium complexes. The quantum global reactivity characteristics, including the electrophilicity index, chemical hardness, chemical softness, ionization potential, and electron affinity, were calculated using the energies of the *HOMO* and *LUMO* frontier orbitals.

***Biomedical investigations***

***Antimicrobial bioassay***

*In vitro* antibacterial potency of the ruthenium complexes was tested against three bacteria strains namely *Escherichia coli* and *Serratia marcescens* as Gram-negative bacteria and *Micrococcus luteus* as Gram-positive bacteria. The tests were done utilizing paper disc diffusion method **[2]**. The nutrient agar medium and 5 mm paper discs were utilized. The complex was dissolved in DMSO and solutions of concentrations 15 and 30 (mg/ml) were prepared. The paper discs were impregnated in various solutions of the specified complex, then dried, and set in Petri plates (9 cm diameter), which were previously seeded with microorganisms. The plates were incubated for 25-30 h at 25 ± 1 °C and the inhibition zones were measured around each disc. Comparison of the obtained data was performed relative to Ofloxacin antibacterial drug.

The antifungal activities of the synthesized complexes against *Geotrichum candidum*, *Aspergillus flavus* and *Fusarium oxysporum* fungi were evaluated using potato Dextrose Agar as a medium. Fluconazole drug was used as standard. In a typical process, a well was performed with the assistance of borer on the nutrient medium plate, which was injected with microorganisms. The well was full of several concentrations of experiment solution using a micropipette and done at 37 °C for 48 h. The tested compounds were dissolved in DMSO making solutions with concentrations of 15 and 30 mg/ml. The activity was observed by measuring the obvious zone of inhibition around the wells. The zone of inhibition was estimated in millimeters (mm).

***Anticancer activity of the complexes***

Three human cancer cell lines were utilized for the *in vitro antitumor* screening tests: breast cancer (MCF7), Colon carcinoma (HCT-116) and liver carcinoma cell (HepG2). Cells were distributed in 96-well microliter plates at concentration of 5x10^4^-1x10^5^ cell/well in a fresh medium and left to bind to the plates for 24 h. Growth inhibition of cells was estimated spectrophotometrically utilizing the standard route with the protein-binding dye sulfa-rhodamine B (SRB). The optical density (OD) of each well was measured at 564 nm with an ELIZA microplate reader (Meter Tech. R 960, USA). The sensibility of the human tumor cell lines was evaluated by the SRB assay. The percentage of cell survival was evaluated as follows:

**Survival fraction = OD (treated cells) / OD (control cells)**

The efficiency of the tested compound was concluded from the IC_50_ value, which is the concentration required to cause 50% inhibition of cell growth. The results were matched with an identical run of Vinblastine as a standard antitumor drug. Various concentrations of the compounds investigated in DMSO (0, 1, 2.5, 5 and 10 μM) were added to the cell monolayer. The monolayer cells were incubated with the complex for 48 h at 37 °C under atmosphere of 5% CO_2_. After then, cells were fixed, rinsed, and stained with Sulforhodamine B stain. The excess stain was washed with acetic acid, while the attached stain was treated with *Tris* EDTA buffer.


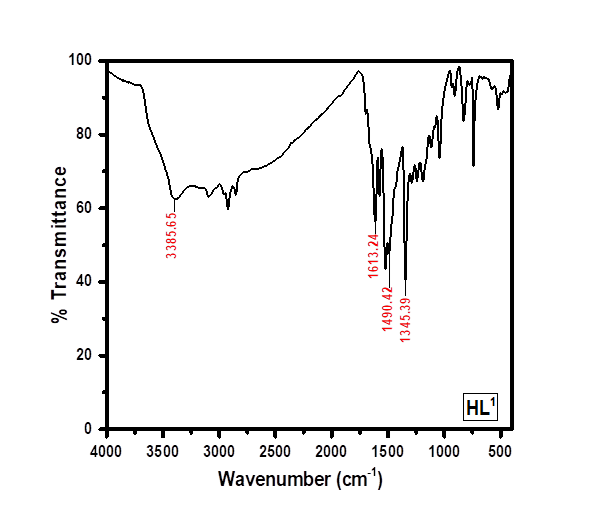


**Figure 1S.** FT-IR spectrum of **HL^1^** ligand.


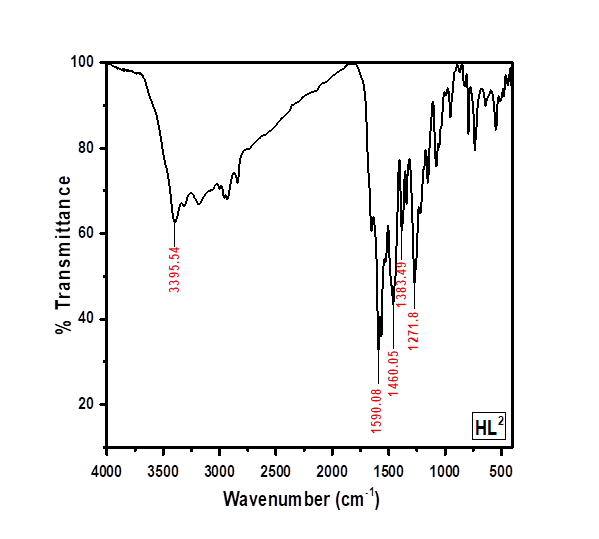


**Figure 2S.** FT-IR spectrum of **HL^2^** ligand.


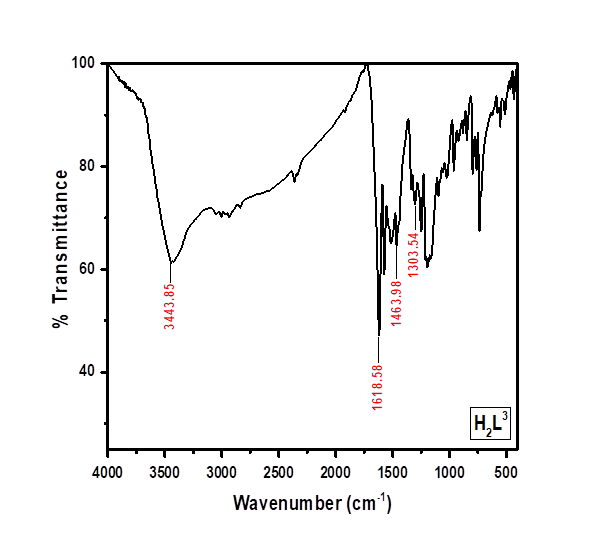


**Figure 3S.** FT-IR spectrum of **H_2_L^3^** ligand.


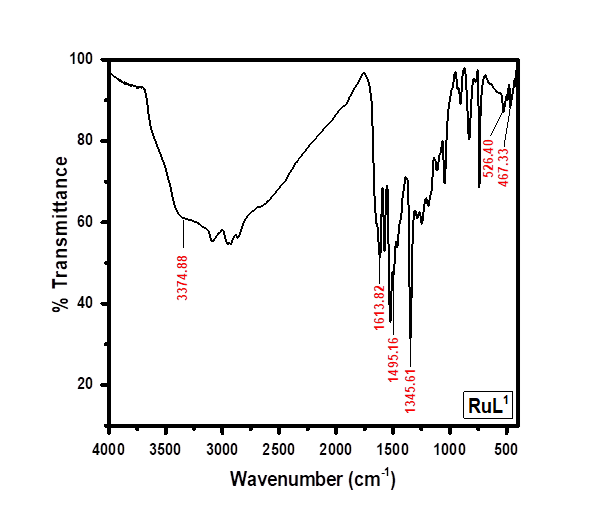


**Figure 4S.** FT-IR spectrum of RuL^1^ complex (**1**).


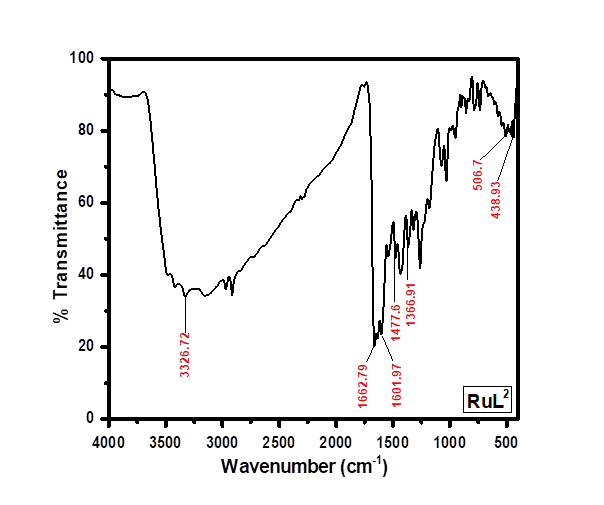


**Figure 5S.** FT-IR spectrum of RuL^2^ complex (**2**).


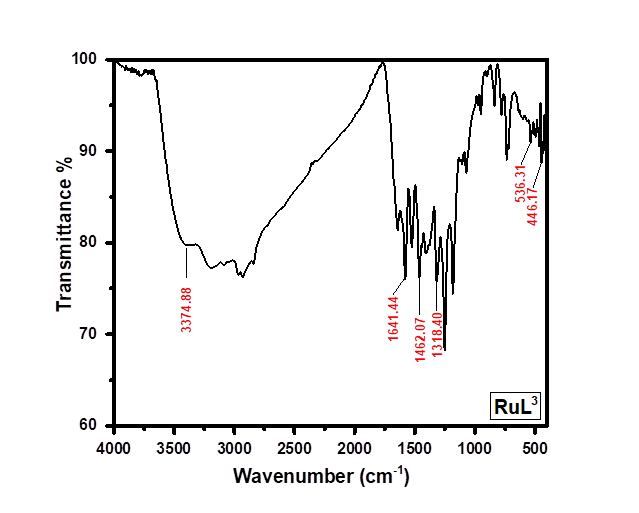


**Figure 6S.** FT-IR spectrum of RuL^3^ complex (**3**).


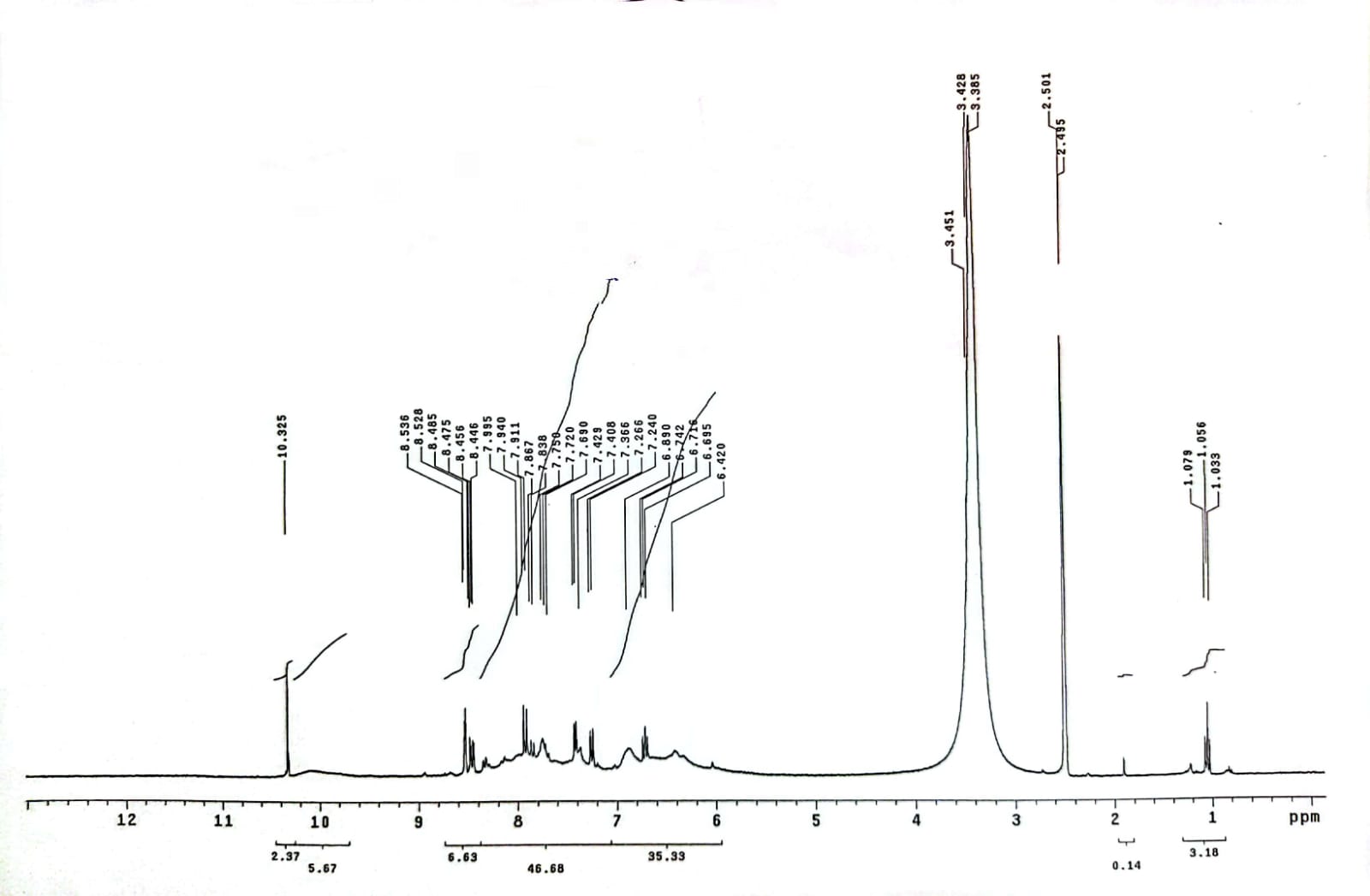


**Figure 7S.** ^1^H NMR spectrum of **HL^1^** ligand.


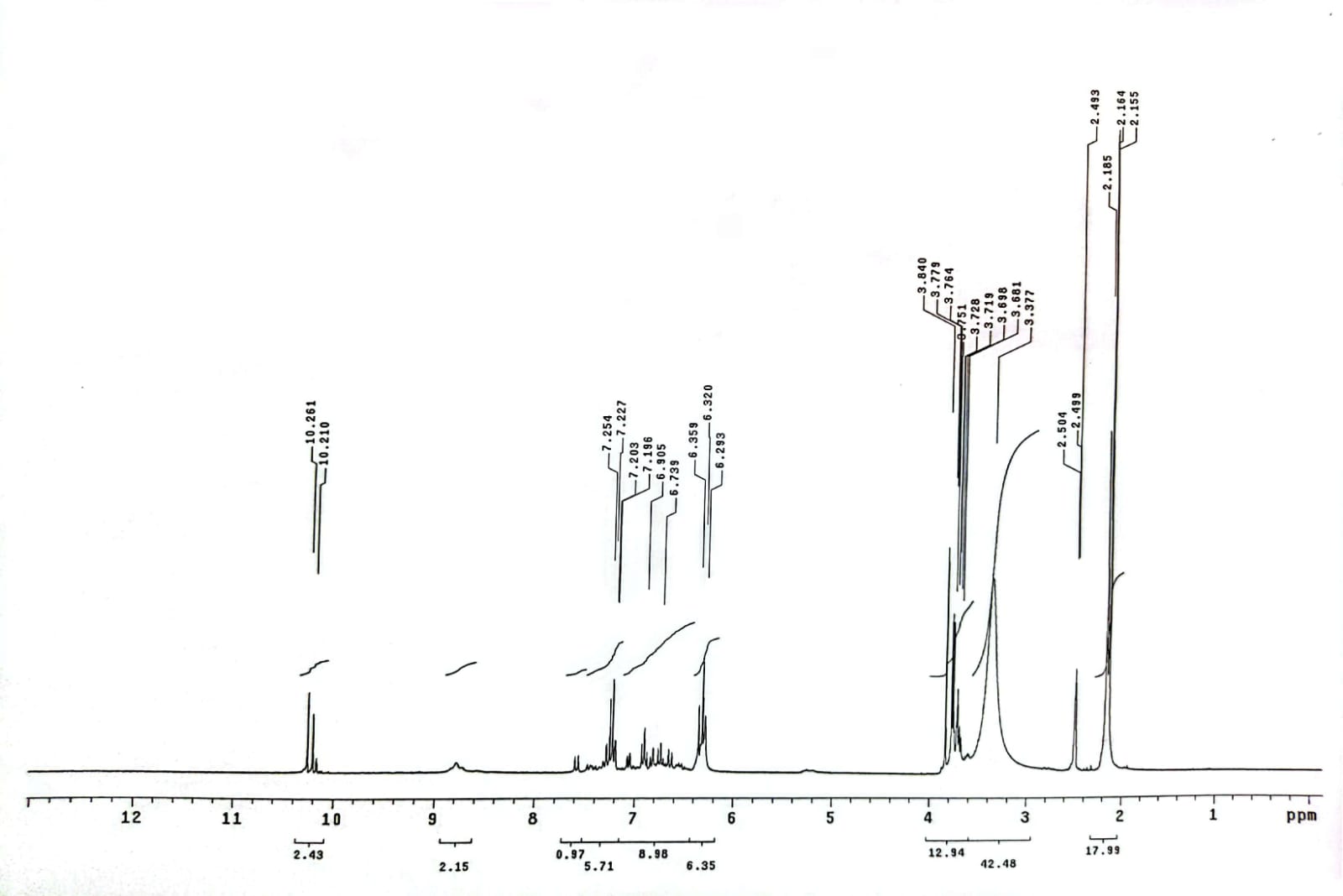


**Figure 8S.** ^1^H NMR spectrum of **HL^2^** ligand.


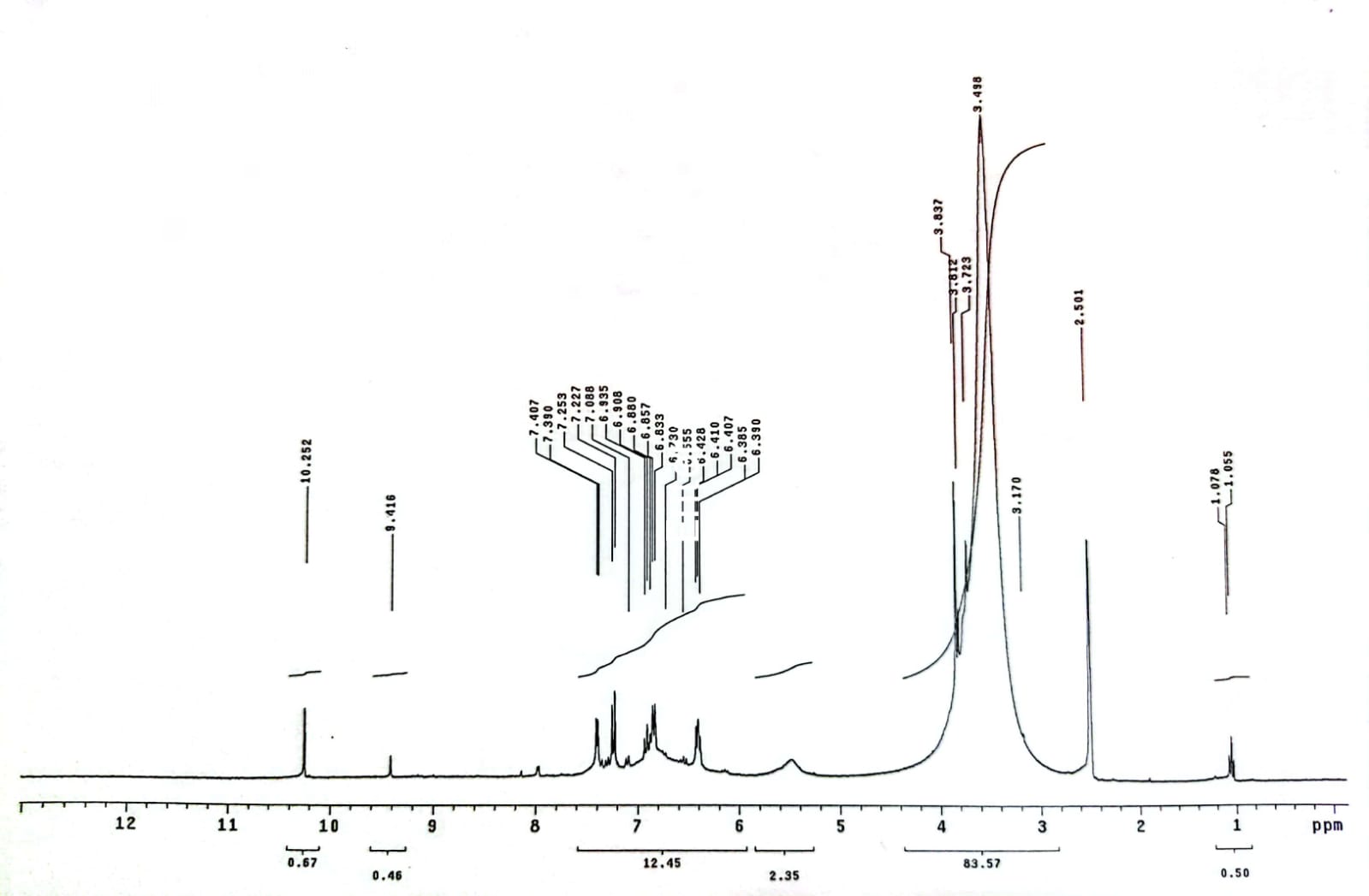


**Figure 9S.** ^1^H NMR spectrum of **H_2_L^3^** ligand.


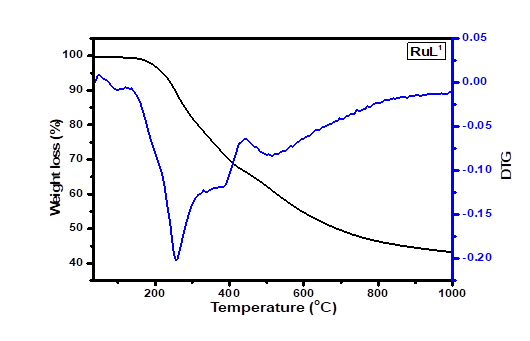


**Figure 10S.** TG and DTG plots of RuL^1^ complex **(1)**.


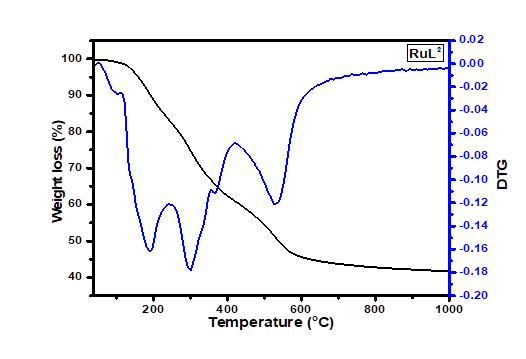


**Figure 11S.** TG and DTG plots of RuL^2^ complex **(2)**.


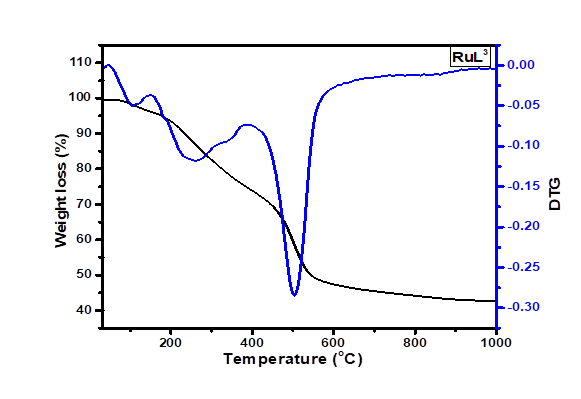


**Figure 12S.** TG and DTG plots of RuL^3^ complex **(3)**.

**Reference**

[1] M. Frisch, G. Trucks, H. Schlegel, G. Scuseria, M. Robb, J. Cheeseman, G. Scalmani, V. Barone, B. Mennucci, G. Petersson, Gaussian 09 Revision A. 1, 2009, Gaussian Inc. Wallingford CT, 139 (2009).

[2] B. Bonev, J. Hooper, J. Parisot, Principles of assessing bacterial susceptibility to antibiotics using the agar diffusion method, Journal of antimicrobial chemotherapy, 61 (2008) 1295-1301.
